# Distinct ways by which Srs2 helicase and Rrp1 translocase shape Rad51–DNA interactions in fission yeast

**DOI:** 10.1093/nar/gkag978

**Published:** 2026-10-13

**Authors:** Dorota Misiorna, Dorota Dziadkowiec, Karol Kramarz

**Affiliations:** Department of Biotransformation, Faculty of Biotechnology, University of Wrocław, Wrocław 50-383, Poland; Department of Biotransformation, Faculty of Biotechnology, University of Wrocław, Wrocław 50-383, Poland; Department of Genetics and Cell Physiology, Faculty of Biological Sciences, University of Wrocław, Wrocław 50-328, Poland

## Abstract

Precise regulation of homologous recombination is essential for genome stability. Multiple helicases and translocases prevent toxic, unscheduled association of the Rad51 recombinase with chromatin, and their levels are frequently dysregulated in cancer. It remains unclear how anti-recombinogenic enzymes act on distinct pools of Rad51. Using *Schizosaccharomyces pombe*, we show that the Srs2 helicase and Rrp1 translocase may target distinct Rad51 species through recombinase substrate specificity and activity control. Genetic analyses support a model in which Srs2 may preferentially regulate Rad52-dependent Rad51 nucleofilaments associated with single-stranded DNA (ssDNA), whereas Rrp1 may preferentially regulate a distinct Rad51 population associated with double-stranded DNA. Accordingly, Rrp1 can alleviate the toxicity caused by Rad51 overproduction, a function not shared by Srs2. Unexpectedly, both loss and overproduction of Srs2 promote Rad51 accumulation, suggesting that excessive helicase levels may generate ssDNA substrates that promote Rad51 nucleation. Both Srs2 and Rrp1 fail to remove strand-exchange-deficient Rad51 from DNA. Both enzymes restrict Rad51 occupancy at centromeric regions, whereas only Srs2 acts within ribosomal DNA (rDNA) repeats, revealing locus-specific regulation. Our findings support a model in which anti-recombinogenic enzymes differentially regulate Rad51 according to its DNA substrate, thereby contributing to a regulatory system that balances Rad51 functions during DNA repair across different genomic regions.

## Graphical Abstract

**Graphical Abstract:**
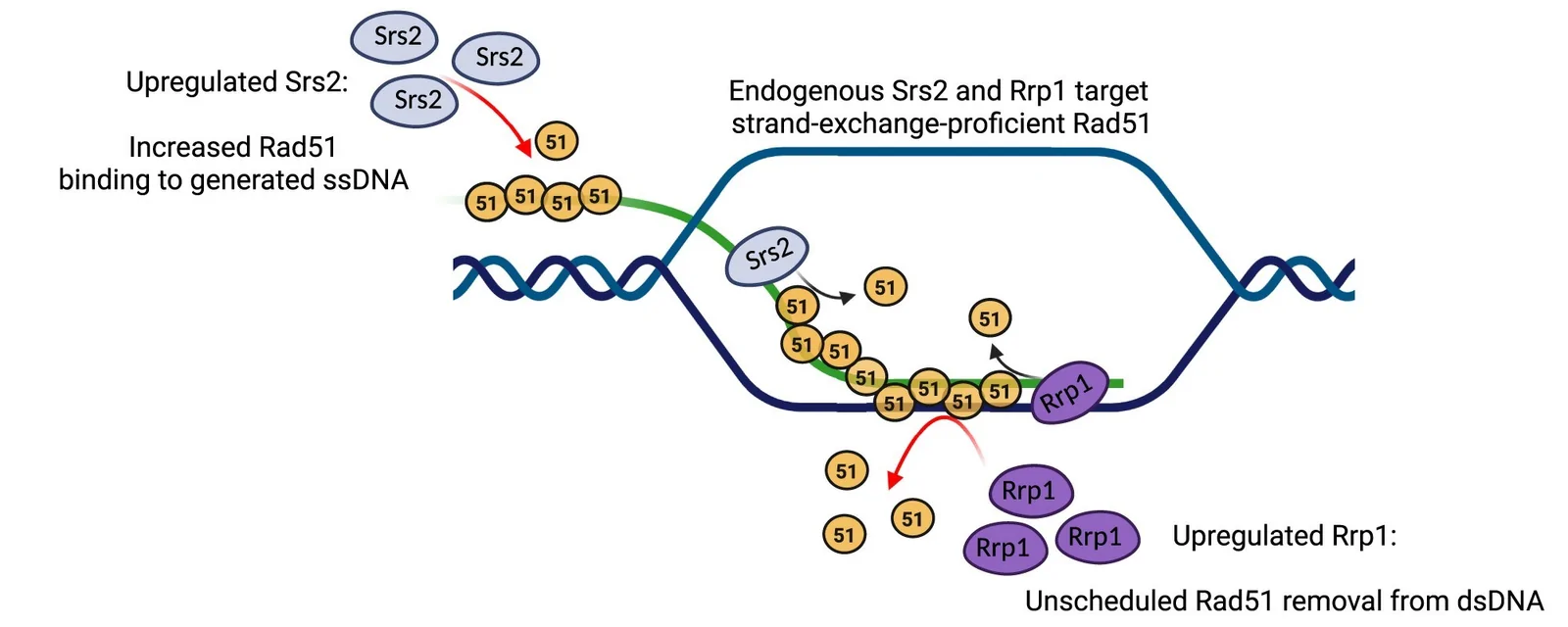

## Introduction

Homologous recombination (HR) is one of the key DNA repair pathways, crucial for the maintenance of genome integrity, and conserved in all eukaryotes [1]. Initially, it was described as the pathway that repairs DNA double-strand breaks (DSBs) using homologous DNA fragments. HR starts with DNA end resection, performed by exonucleases generating 3′ single-stranded DNA (ssDNA) overhangs coated immediately by replication protein A (RPA). In fission yeast, Rad52 is the main HR mediator that facilitates the replacement of RPA with Rad51 [2]. The Rad51 recombinase is a central player in HR that forms nucleofilaments on ssDNA to perform a homology search in the genome. Several auxiliary proteins (Rad55–Rad57, Swi5–Sfr1, and Rad54) facilitate the strand exchange reaction mediated by Rad51 nucleofilament involving invasion into the homologous duplex DNA and formation of a displacement loop (D-loop). Subsequently, Rad51 is dissociated from heteroduplex DNA ends by Rad54 translocase, allowing for polymerase binding and initiation of DNA synthesis that extends the D-loop. The final step entails either the displacement of newly synthesized DNA strand by various helicases in a process termed the synthesis-dependent strand annealing (SDSA) or, after D-loop extension and second-end capture, the resulting double Holliday junction is processed by relevant endonucleases and/or helicase–topoisomerase III complexes [1, 2]. In human cells HR proceeds through corresponding major steps but requires many additional regulatory proteins such as BRCA1 to promote end resection and assist BRCA2/PALB2 loading to mediate RAD51 assembly on ssDNA [3]. Other accessory factors such as RAD51 paralogs are also involved in stabilization of the RAD51 nucleofilament in human cells [4].

RAD51 alone has similar affinity for ssDNA, crucial for HR, and double-stranded DNA (dsDNA), indispensable for replication fork protection and reversal [5, 6]. Stalled replication forks, apart from being protected by RAD51, might be restarted by a RAD51-dependent process [7]. Excessive HR due to aberrant RAD51 loading on ssDNA may lead to chromosome instability [8, 9], while RAD51–dsDNA complexes inhibit DNA pairing during HR in budding yeast [10]. Additionally, as mentioned earlier, RAD51 also needs to be timely removed from heteroduplex DNA before DNA synthesis can be initiated during HR repair. Elevated levels of RAD51 are detrimental for cells, as has been demonstrated in yeast and humans [11, 12]. This is also clearly visible in fission yeast cells that become elongated and suffer viability loss upon *rad51+* overexpression [13]. RAD51 was also found to be overexpressed in multiple tumours, especially those harbouring a p53 mutation [14]. Overall, it is now believed that RAD51-dependent HR might actually drive cancer progression and contribute to therapy resistance [15]. This underscores the need for tight regulation of RAD51 binding to and/or its timely removal from both ss- and dsDNA to prevent inappropriate recombination events and inadequate protection of replication forks and thus preserve genome stability.

One of the best-described DNA helicases involved in Rad51 regulation is budding yeast Srs2 (referred to as scSrs2). It is a 3′–5′ DNA helicase that binds to and translocates along ssDNA using ATP hydrolysis and disrupts Rad51 nucleofilaments, which prevents unscheduled recombination events and directs repair into the error-free SDSA pathway [16, 17]. Additionally, scSrs2 functions together with SUMOylated form of the essential DNA replication factor PCNA (proliferating cell nuclear antigen) to channel post-replication repair (PRR) away from HR [18, 19]. Loss of scSrs2 leads to hyperrecombination and sensitizes *srs2Δ* cells to DNA-damaging drugs [20]. Based on those findings, scSrs2 was described as an anti-recombinase, particularly important to inhibit recombination at stalled replication forks. However, it was reported that scSrs2 might also have pro-recombinogenic functions, dependent on its SUMOylation but separate from its interaction with PCNA [21]. Srs2 helicase from *Schizosaccharomyces pombe* is shorter than its budding yeast orthologue and lacks part of the C-terminus containing established Rad51 and PCNA interaction motifs of *sc*Srs2. Deletion of *srs2+* in *S. pombe* leads to decrease in the frequency of HR and results in DNA damage sensitivity [22], but cannot suppress DNA damage sensitivity of mutants in PRR pathway [19, 23, 24]. This indicates that *S. pombe* Srs2 may have different cellular functions than its *S. cerevisiae* counterpart. Exploring them in more detail is important since human cells do not contain a direct Srs2 orthologue. Instead, several helicases and translocases, such as WRN, PARI, and FBH1, have been suggested to play similar anti-recombinogenic roles [25]. FBH1, e.g. was proposed to remove RAD51 from ssDNA and suppress HR [26]. Fbh1 is not present in budding yeast but was described in *S. pombe* and shown to translocate on ssDNA to remove Rad51. When overexpressed, Fbh1 reduced recombination-dependent replication and contributed to the global reduction of Rad51 foci induced by replication stress-generating drugs [27].

The Swi2/Snf2 family DNA translocase Rad54 has several important functions in the stimulation of Rad51 during HR as described earlier. Rad54 also translocates along dsDNA [28], assisting in Rad51-mediated homology search. However, Rad54 translocase is also crucial to prevent unscheduled and excessive Rad51 binding to dsDNA in yeast and human cells [11, 12]. Interestingly, Rad54 restricts the anti-recombinase activity of Srs2 in *S. cerevisiae* [32]. Accordingly, we have previously shown that Uls1, a budding yeast DNA translocase and SUMO-targeted ubiquitin ligase, was involved in the suppression of a subset of Rad51-dependent HR pathways at replication forks stalled on endogenous obstacles by downregulating the levels of SUMOylated scSrs2 [30]. Uls1 was also shown to restrict Rad51 binding to dsDNA together with Rad54 [11]. In fission yeast, Rad54 was recently reported to prevent excessive Rad51 accumulation during the repair of spontaneous DNA breaks arising in S phase [31]. Rrp1, an orthologue of Uls1 in fission yeast, has been reported to function with Srs2 in a Swi5–Sfr1-dependent HR sub-pathway [22]. Interestingly, its ability to remove Rad51 from dsDNA in an ATPase-dependent manner, confirmed by biochemical analyses, was essential to alleviate the toxicity induced by Rad51 overproduction [13].

Thus, regulation of Rad51 chromatin binding in both yeasts and humans involves interactions between similar helicases and translocases to minimize the risk of genomic rearrangements. This may also depend on the source of DNA damage or replication stress, the DNA template itself (ss- or dsDNA), or specific chromatin loci (e.g. centromeres or rDNA). Various helicases, such as Srs2, Fbh1, and others, as well as translocases, such as Rad54, Uls1, and Rrp1, may be differentially employed in these situations to allow specific but comprehensive regulation of Rad51 activity. Interestingly, the expression of many DNA helicases, including FBH1, is upregulated in several types of cancer and required for their proliferation and resistance to DNA damage [32, 33].

In this work, we thus aimed to better characterize spSrs2 and Rrp1 as Rad51 regulators in *S. pombe* with emphasis on the effects of their upregulation on Rad51 chromatin occupancy. We provide genetic evidence consistent with the idea that spSrs2 may preferentially regulateRad51 nucleofilaments assembled on ssDNA. Interestingly, spSrs2 can also promote Rad51 nucleation, in contrast to its budding yeast orthologue. We propose that even though spSrs2 and Rrp1 interact and are present at similar sites in the nucleus, they seem to regulate Rad51 independently of each other. Rrp1 cannot target Rad51 accumulating when spSrs2 is absent or overproduced, suggesting it only engages with Rad51 bound to dsDNA. Additionally, Rrp1, as opposed to spSrs2, is not involved in ensuring proper rDNA replication. Finally, our microscopic analysis of Rad51 foci suggests that spSrs2 and Rrp1 cannot remove strand-exchange-deficient Rad51 from DNA, but unexpectedly, Rrp1 rather stabilizes it, in a process possibly dependent on Rad51 ubiquitination.

## Materials and methods

### Yeast strains, plasmids, and general methods

Strains, plasmids, and primers used in this study are listed in Supplementary Tables S1 and S2. Standard media were used for *S. pombe* propagation [34]. Yeast cells were grown in complete yeast extract plus supplements (YES) medium or glutamate-supplemented Edinburgh minimal medium (EMMg). Thiamine at 5 μg/ml, geneticin (ICN Biomedicals) at 100 μg/ml, and nourseothricin (Werner Bioagents) at 200 μg/ml were added where required. Multiple mutants were obtained by genetic crossing of relevant single mutants followed either by random spore analysis or by tetrad dissection. The pREP81-FLAG plasmid carrying wild-type (WT) *srs2^+^* was constructed using the Gibson Assembly® Cloning Kit (NEB E5510S) and cloned into other required plasmids as described in [35]. pREP81-FLAG containing *S. cerevisiae SRS2* gene was prepared by standard ligation of polymerase chain reaction (PCR)-amplified sc*SRS2* gene. pREP81-FLAG containing a truncated spSrs2, devoid of last 10 amino acids (Srs2-CΔ, 1–877), was prepared by standard ligation of PCR-amplified truncated *S. pombe srs2^+^*gene from *pREP81-srs2-FLAG*. In all cases, expression of relevant proteins by *nmt* promoters was induced after 24 h growth in minimal media without thiamine.

### Spot assays

Cells were grown to mid-log phase, then serially 10-fold diluted, and 2 μl aliquots were spotted onto relevant plates (YES or EMMg) either without drugs or containing camptothecin (CPT) or hydroxyurea (HU). Plates were incubated for 3–5 days at 30°C and photographed. All assays were repeated at least twice.

### Growth rate measurements

Strains with relevant plasmids were pre-cultured in 20 ml of EMMg supplemented with thiamine (to suppress expression from the *nmt* promoter). Then cells were washed with EMMg and inoculated into fresh EMMg media without thiamine (to induce expression from *nmt* promoter) at initial OD_600_ = 0.01. The prepared inoculum was redistributed into wells of a 96-well plate (Sarstedt, Nümbrecht, Germany) and incubated for 70 h at 28°C with continuous orbital shaking (5 mm amplitude and 120 rpm frequency) in a Spark microplate reader and incubator (Tecan, Männedorf, Switzerland). OD_600_ was measured every 1 h. EMMg medium alone served as a negative control of the experiment. Each strain/transformant was analysed in triplicate, and the experiment was repeated two times.

### Live-cell imaging

Cells were grown in YES or EMMg medium (with or without thiamine to suppress or induce expression from *nmt* promoter, respectively) to exponential phase, and an aliquot was placed on microscope slide. Nine z-stack pictures (each z-step of 250 nm) were captured using the Axio Imager M1 epifluorescence microscope (Carl Zeiss) equipped with a 100× immersion oil objective (Plan-Neofluar 1006/1.30), the GFP/dsRED filter set, and differential interference contrast (DIC). Images were acquired with AxioCam MRc digital colour camera and ZEN 3.7 software (Carl Zeiss). Pictures were processed and analysed with ImageJ.

### Super-resolution microscopy

Cells were grown in EMMg medium without thiamine to induce *nmt* promoter until they reached exponential phase, and an aliquot was deposited onto microscope slide covered with a thin layer of 1.4% agarose resuspended in EMMg. Images were acquired on a ZEISS Elyra 7 microscope using a Plan-Apochromat 63×/1.4 Oil DIC M27 objective with oil immersion. The experiment was conducted at a constant temperature of 30 °C. Fluorescence was excited using 488 nm (HR Diode 488 nm 100 mW, 10% fixed power range) and 561 nm (HR DPSS 561 nm 500 mW, 50% fixed power range) laser lines. Images were captured on a CameraMonochrome2 sCMOS detector at 1024 × 1024 pixels, 16–bit depth, and 6.45 nm pixel size. Image acquisition was performed in structured illumination SIMFast mode with 13 phases per plane of a 27.5 µm grid period for 488 nm and 32 µm period for 561 nm, using SIM^2^ reconstruction. Images were processed using the SIM^2^ algorithm implemented in the ZEISS acquisition software, using default diffraction efficiency settings for each channel. Pictures were then processed using ImageJ.

### Whole protein extract analysis

The trichloroacetic acid (TCA) method was used to isolate protein extracts from logarithmic cultures in EMMg without thiamine to induce *nmt* promoter. Twenty millilitres of culture (OD_600_ ∼1) was collected and resuspended in 300 μl of water. Then, cells were mixed with 350 μl of freshly prepared lysis buffer (2 M NaOH, 7% β-merkaptoethanol) and 350 μl of 50% TCA. After centrifugation, pellets were further washed with 1 M Tris–HCl, pH 8, and resuspended in 2× Laemmli buffer (62.5 mM Tris–HCl, pH 6.8, 20% glycerol, 2% sodium dodecyl sulphate (SDS), 5% β-mercaptoethanol with bromophenol blue). Samples were boiled before being subjected to SDS–polyacrylamide gel electrophoresis (PAGE) on Mini-PROTEAN TGX Precast Gel 4%–15% (Bio-Rad, 4561086). Western blots were performed with following antibodies at relevant dilutions: 1:1000 anti-GFP (Roche, 11814460001), 1:3000 anti-HA (Sigma–Aldrich, H6908), 1:5000 anti-Rad51 (Abcam, ab63799), 1:2000 anti-FLAG (Sigma–Aldrich, F7425), 1:500 anti-ubiquitin (P4D1, Santa Cruz Biotechnology, sc-8017), and anti-PCNA (PC10, Santa Cruz Biotechnology, sc-56). Ponceau S staining of blotted membranes was used as loading controls. Experimental details are provided in relevant figure captions. All uncropped western blots presented across the figures are shown in Supplementary Figs S10–S12.

### *In vivo* co-immunoprecipitation

Cells were grown to exponential phase using EMMg medium without thiamine to induce *nmt* promoter for 24 h. Next, 150 ml of cells were harvested and broken with glass beads in 400 μl of ice-cold lysis buffer [50 mM HEPES–KOH, pH 7.5, 100 mM KCl, 2.5 mM MgCl_2_, 10% glycerol, protease inhibitors (Sigma–Aldrich, P8215), 1 mM PMSF]. Extracts were cleared by centrifugation for 15 min at 15 000 rpm. To digest nucleic acids, lysates were incubated with 500 U of Benzonase (Sigma–Aldrich, E8263) for 1 h at 4°C on a rotator wheel. To capture FLAG-tagged proteins, lysates were incubated with DYKDDDDK Fab-Trap® (ChromoTek, ffa) overnight at 4°C. The beads were subsequently washed four times with lysis buffer followed by protein elution with Laemmli buffer (2% SDS, 20% glycerol, 120 mM Tris, pH 6.8, 4% β-mercaptoethanol, 0.01% bromophenol blue). Proteins were resolved on Mini-PROTEAN TGX gels (Bio-Rad, 4561096). Fifty microlitres of whole-cell extract used for immunoprecipitation was analysed as input. For detection, anti-FLAG (Sigma–Aldrich, F7425), anti-GFP (Invitrogen, A11122), and anti-PCNA (PC10, Santa Cruz, sc-56) antibodies, and anti-Rad51 [13, 36] antiserum were used.

### Rad51 and Rad11 immunofluorescence

Logarithmic strains grown in EMMg medium (without thiamine to induce expression from plasmids) were fixed with 4% formaldehyde for 20 min at room temperature (RT). The excess of formaldehyde was removed by washing with phosphate buffered saline (PBS) and PEM buffer (PEM: PIPES-EGTA-MgSO₄: 100 mM PIPES, 1 mM EGTA [ethylene glycol-bis(β-aminoethyl ether)-N,N,N′,N′-tetraacetic acid], 1 mM MgSO_4_, pH 6.9). From that moment, all centrifugations were done for 5 min at 600 × *g*. Spheroplasts were obtained by digestion of cells for 10 min at 30°C with Zymolyase 100T (BioShop, ZYM002) at concentration 0.5 µg/ml in PEMS (PEM with 1.2 M sorbitol). Zymolyase was removed by three washes with PEMS, and then cells were exposed to 1% Triton X-100 in PEMS for 5 min, RT. Cells were then washed two times with PEMBAL [1% bovine serum albumin, 0.1% sodium azide, 100 mM lysine monohydrate (Sigma–Aldrich, L-5626) resuspended in PEM buffer] and then incubated for 1 h on the rotation wheel in PEMBAL at RT. Then the cells were resuspended in 300 μl of PEMBAL with anti-Rad51 antibody (Abcam, ab63799, 1:300) and, in case of double immunofluorescence, additionally with anti-GFP (Roche, 11814460001, 1:300). Cells were then incubated overnight at 20°C. Next morning, three washes with 1 ml of PEMBAL were done—two for 10 min and the last one for 30 min on the rotation wheel in PEMBAL at RT. Then cells were resuspended in 300 μl of PEMBAL with anti-Rabbit Alexa Fluor 555 (Invitrogen, A21428, 1:2000) and, in case of double immunofluorescence additionally with anti-Mouse Alexa Fluor 488 (Invitrogen, A-11001, 1:2000). Cells were then incubated on the wheel at RT for 1.5 h at RT. After that time, three washes with 1 ml of PBS were done, each for 10 min, and then cells were resuspended in 1 ml of PBS with DAPI (1:4000) for 3 min. Next, a brief wash with PBS was done to remove the excess of DAPI. Lastly, cells were resuspended in 10 μl of ProLong Gold antifade reagent (Invitrogen, P36934) and imaged using Axio Imager M1 microscope (Carl Zeiss). Nine z-stack pictures of 250 nm were collected with 300 ms exposure for Alexa 555/488 and 20 ms for DAPI channels using ZEN 3.7 software (Carl Zeiss). Images were analysed using ImageJ.

### EdU assay

Yeast cells bearing the human equilibrative nucleoside transporter (hENT1) and the *Drosophila melanogaster* nucleotide (thymidine) kinase (dmNK) were grown in EMMg not supplemented with amino acids an without thiamine to induce expression of plasmids until early exponential phase, and exposed to a short pulse of the thymidine analog 5-ethynyl-2′-deoxyuridine (EdU) (Invitrogen, A10044) added to each culture to a final concentration of 50 μM for 10 min at RT. Cells were fixed in ethanol 70% and washed once in PBS and twice in PBS with 1% Triton. Cells were resuspended in 200 μl of freshly made Click-iT solution: 100 mM sodium ascorbate, 2 mM CuSO_4_, 0.5 μM Alexa Fluor 488 azide (Invitrogen, A10266) in PBS. After a 1 h incubation on a wheel at RT in the dark, for imaging, cells were centrifuged, washed in 1 ml of PBS, and resuspended in 1/20 the original volume. A drop was applied onto a microscope slide, covered with a coverslip, and viewed in fluorescence microscope. Nine z-stack images (step size of 0.25 μm) were collected using Axio Imager M1 microscope (Carl Zeiss). Images were analysed with Fiji software. Non-S-phase cells with EdU signal were distinguished by the absence of septum.

### Two-dimensional gel electrophoresis analysis of rDNA locus

Analysis of replication profile within rDNA locus was performed as described earlier [35] with following modifications. Logarithmic cultures (2.5 × 10^9^ cells per sample) were treated with 0.1% sodium azide and then 50 ml of 0.5 M ethylenediaminetetraacetic acid (EDTA), pH 8, was added to each culture. Cells were exposed to trimethyl psoralen (0.01 mg/ml TMP, Sigma, T6137) for 5 min in the dark and subsequently irradiated with UV-A (365 nm), 10 mW/cm^2^ for 8 min. Next, samples were digested with lysing enzymes (Sigma, L1412, 0.625 mg/ml) and Zymolyase 100T (Amsbio, 120493–1, 0.5 mg/ml). Spheroplasts were directly digested for 30 min with RNAse (Roche, 11119915001) in G2 buffer (800 mM guanidine hydrochloride; 30 mM Tris–HCl, pH 8.0; 30 mM EDTA, pH 8.0; 5% Tween 20; 0.5% Triton X-100 with 1.5% *N*-lauryl sarcosine) and subsequently, for 1 h at 55°C with 1mg/ml proteinase K. Genomic DNA was recovered by applying the samples on QIAGEN Genomic-tip 100/G columns (Qiagen, 10243) according to manufacturer’s instructions. Total DNA was precipitated overnight with isopropanol in −20°C. Precipitated DNA was washed with 70% ethanol and digested with 500 units per sample of *BamHI* (NEB, R0136T) for 3 h. Extracted genomic DNA with replication intermediates was migrated in 0.4% ultrapure agarose gel in 1× TBE (Tris, Borate, EDTA [Ethylenediaminetetraacetic acid] buffer) for first dimension. The slices of DNA relevant for *BamHI* 3.1 kB fragment of rDNA were cast for second dimension in 1% ultrapure agarose gel in presence of 0.5 µg/ml ethidium bromide. The DNA was capillary-transferred onto neutral nylon membrane (GE Healthcare, RPN203N) in 10× SSC (Saline Sodium Citrate). Membranes were incubated with 1354 bp rDNA probes (amplified with primers listed in Supplementary Table S2). The chemiluminescent kit AlkPhos Direct Labelling Module (Cytiva, RPN3680) with CDP-Star detection reagent (RPN3682) was used for Southern blot detection. The signal of replication intermediates was collected in a ChemiDoc MP (Biorad).

### Chromatin immunoprecipitation

Strains transformed with pREP41-EGFP-Srs2-FLAG or pREP41-Rrp1-FLAG were subjected to chromatin immunoprecipitation (ChIP) as described in [35]. Rad51 ChIP was performed as described earlier [37]. Both ChIPs were done with the following modifications. One hundred millilitres of exponential culture (OD600 ∼1) was crosslinked with 1% formaldehyde (Sigma, F-8775). Cells were frozen in liquid nitrogen and extracted in lysis buffer [50 mM HEPES, pH 7.5, 1% Triton X-100, 0.1% deoxycholate, 1 mM EDTA with 1 mM PMSF and protease inhibitors (Sigma–Aldrich, P8215)]. Chromatin sonication was done in a Diagenode Bioruptor Pico using Easy mode: 10 cycles of 30 s ON and 30 s OFF. Overnight immunoprecipitation was performed as follows: 300 μl of lysate was incubated with anti-FLAG (Sigma–Aldrich, F7425) or anti-Rad51 [13, 36] antiserum at 1:150 concentration, and 5 μl was preserved as the INPUT fraction. After incubation, Protein G Dynabeads (Invitrogen, 10003D) were added for 2 h, and immunoprecipitated complexes were decrosslinked overnight at 65°C. The DNA associated with Srs2 or Rrp1 was purified with a Qiaquick PCR purification kit (QIAGEN, 28104) and eluted in 300 μl of water. DNA associated with Rad51 was precipitated with isopropanol/glycogen/3 M potassium acetate for 1 h at −20°C. Precipitated DNA was centrifuged at 15 000 rpm for 20 min at 4°C and then washed with cold 70% ethanol. After centrifugation, the DNA pellet was resuspended in 300 μl of Nuclease-Free Water (Sigma–Aldrich, W4502). Quantitative polymerase chain reaction (SsoAdvanced Universal SYBR® Green Supermix, #1725274; primers listed in Supplementary Table S2) was performed to determine the Ct using Bio-Rad CFX Maestro v1.1. Enrichment for Rad51, Srs2, and Rrp1 was calculated as % of input normalized by subtraction values obtained for the intergenic locus on chromosome II. For Rad51 ChIP, values calculated for *rad51Δ* strain (a negative control) were subtracted to obtain final, normalized Rad51 enrichment as % of input.

### Ni-NTA pulldown

Ni-NTA pulldown was done as described in [38]. Logarithmic strains were grown in EMMg without thiamine. One hundred millilitres of culture (OD_600_ ∼1) was harvested by centrifugation, washed once with water, and resuspended in 450 μl of buffer G (100 mM sodium phosphate, pH 8.0; 10 mM Tris–HCl, pH 8.0; 6 M guanidine hydrochloride) with 1% Triton X-100, protease inhibitor cocktail (Sigma–Aldrich, P8215), 1 mM PMSF, and 20 mM *N*-ethylmaleimide. Cells were lysed by bead beating (4 × 30 s at 6500 rpm with 3 min intervals on ice). Lysates were cleared by centrifugation at 18 000 × *g* for 20 min at 4°C, and 20 μl of the supernatant was saved as input. The remaining lysate was applied to Ni-NTA agarose columns (New England Biolabs, S1427L), prewashed with buffer G, and incubated for 1.5 h at 4°C with rotation. After incubation, columns were washed twice with buffer G containing 5 mM imidazole, followed by two washes with buffer U (8 M urea; 100 mM sodium phosphate, pH 6.4; 10 mM Tris–HCl, pH 6.4; 0.1% Triton X-100) with 5 mM imidazole. Proteins were eluted in two steps by addition of 100 μl of IMAC elution buffer (1× IMAC: 0.02 M sodium phosphate, 0.3 M NaCl, pH 7.4, and 500 mM imidazole) with 5 min incubation per step. Eluates were precipitated with an 200 μl of 50% TCA. Input samples were mixed with 80 μl water and 100 μl 50% TCA. All samples were incubated on ice for 20 min and centrifuged at 18 000 × *g* for 20 min at 4°C. Obtained pellets were washed with 1 M Tris–HCl, pH 8.0, centrifuged again, and resuspended in Laemmli buffer (17 μl for IP, 40 μl for input; 62.5 mM Tris–HCl, pH 6.8; 20% glycerol; 2% SDS; 5% β-mercaptoethanol; bromophenol blue). Samples were boiled at 100°C for 5 min and analysed by SDS–PAGE.

### Time-lapse microscopy

*nmt-GFP-Rad51* cells were grown in EMMg medium lacking thiamine for 24 h to induce *GFP-rad51* overexpression. Cells were harvested and resuspended in 500 μl EMMg, and 2 μl of concentrated culture was mounted on Thermo Scientific glass-bottom slides (ER-201B-CE24) coated with a thin layer of 1.4% agarose prepared in filtered EMMg. Time-lapse imaging was performed using a Zeiss Lattice Lightsheet 7 microscope equipped with a 13.3×/0.4 NA illumination objective, a 44.83×/1.0 NA water-immersion detection objective, and a Hamamatsu ORCA-Fusion sCMOS camera. GFP fluorescence was excited using a 488 nm laser (2% laser power) and collected through a 500–588 nm emission filter. Image stacks consisting of 501 optical sections were acquired with a 0.2 μm z-step (voxel size 0.145 × 0.145 × 0.200 μm) every 30 s for 30 min using a 30 ms exposure time, resulting in 61 time points. Images were acquired using ZEN 3.7 (Zeiss).

Raw datasets were deconvolved in ZEN 3.7 using the constrained iterative deconvolution algorithm (12 iterations, Poisson likelihood model) and analysed in Fiji/ImageJ. MAX-intensity z-projections were generated and corrected for photobleaching using the Stack Contrast Adjustment plugin. The resulting 2D time-lapse movies were analysed to assess Rad51 filament dynamics in cells carrying the empty vector or overexpressing *rrp1^+^* or *srs2^+^*. Representative movies are provided as Supplementary Movies 1–3, and the figure panels (Fig. 6F) show montages presenting frames with 6 min intervals for each movie.

### Statistical data analysis

Student’s *t*-test was used to calculate the *P*-values (^****^P ≤ .0001, ***P ≤ .001, ^**^ .001 < *P* ≤ .01, ^*^ .01 < *P* ≤ .05). The numbers of replicates are specified within figure captions were relevant.

## Results

### *Schizosaccharomyces pombe* Srs2 helicase interacts with PCNA and Rad51 like its *S. cerevisiae* orthologue

Alignment of Srs2 protein sequences from *S. pombe* (spSrs2) and *S. cerevisiae* (scSrs2) demonstrates (Fig. 1A) that the *S. pombe* protein is shorter than its *S. cerevisiae* orthologue and lacks the C-terminal fragment containing motifs previously described as important for scSrs2 interactions with Rad51, PCNA (PIM), and SUMO (SIM) [21, 39–42]. However, by sequence analysis, we found a potential PIM at the very C-terminal end of the fission yeast Srs2 and fragments of a region important for Rad51 interaction described for scSrs2 (Fig. 1A and B). Superposition of three-dimensional Srs2 protein structures from *S. pombe* and *S. cerevisiae* obtained using AlphaFold [43] highlights a high degree of conservation of their respective UvrD-like helicase domains (Supplementary Fig. S1A), while *S. pombe* Srs2 potential PCNA and Rad51 interaction domains are located within unstructured regions of the protein and do not align with those of its *S. cerevisiae* counterpart. Nevertheless, we were able to co-immunoprecipitate endogenously expressed native PCNA (Fig. 1C) and Rad51 (Fig. 1D) with overproduced fission yeast Srs2-FLAG, thus demonstrating that PCNA-Srs2 and Rad51-Srs2 complexes may be formed *in vivo* in *S. pombe* cells. PCNA also co-immunoprecipitated with overproduced Srs2-CΔ, a truncated *S. pombe* Srs2, devoid of the potential PIM region (Supplementary Fig. S1B), suggesting that other regions of Srs2 are presumably crucial for PCNA interaction and PIM might play only an auxiliary role.

**Figure 1.**
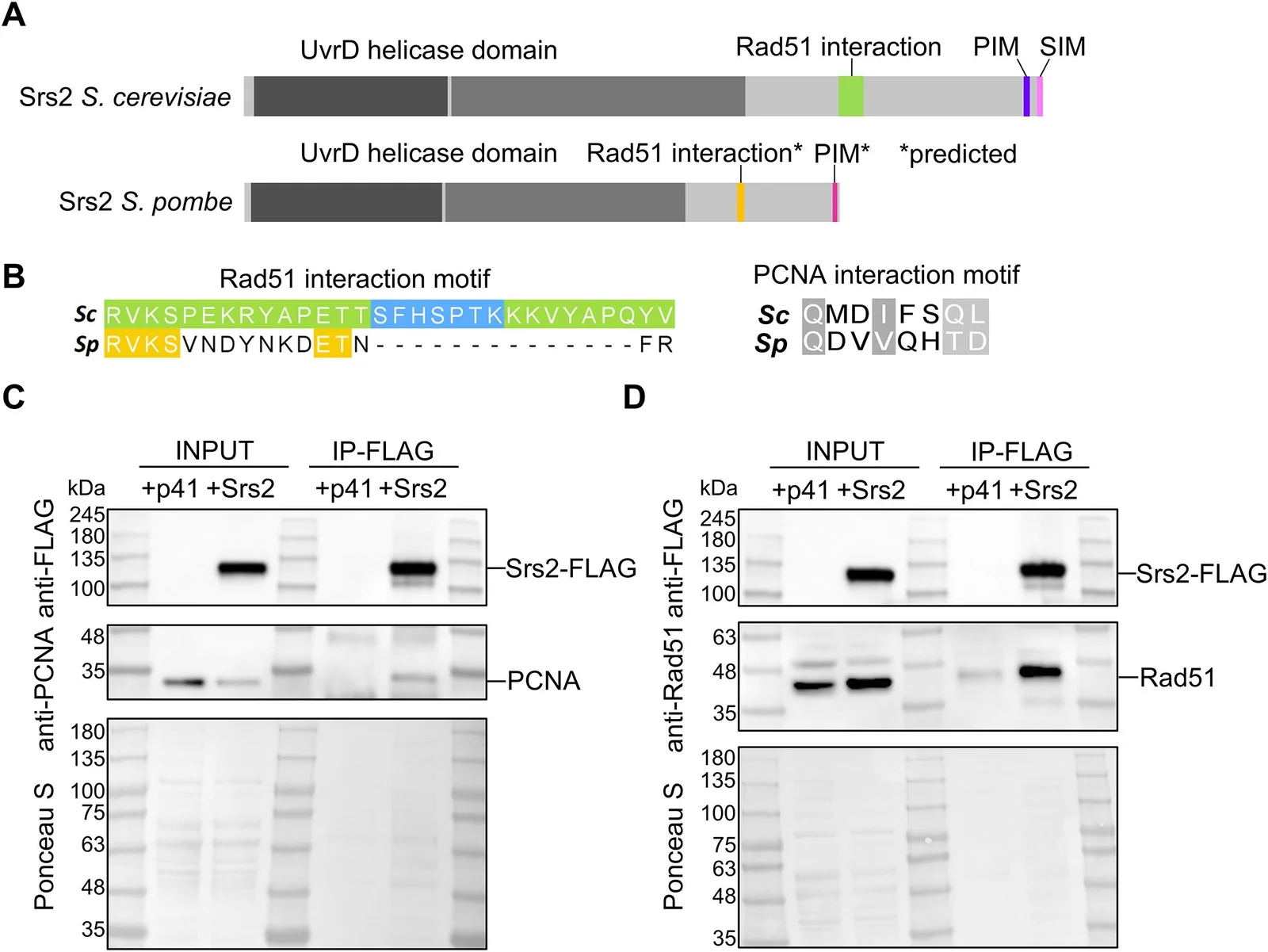
spSrs2 interacts with PCNA and Rad51 in fission yeast similarly to budding yeast scSrs2. (**A**) Scheme depicting characteristic domains and motifs of scSrs2 and spSrs2. PIM – PCNA-interacting motif, SIM – SUMO-interacting motif. Green box – Rad51 interaction domain. Orange box – predicted Rad51 interaction domain. Grey box – UvrD helicase N- and C-terminal domain. (**B**) Alignment of Rad51 interaction motif between scSrs2 and spSrs2. (**C**) Co-immunoprecipitation of spSrs2-FLAG and endogenous PCNA. WT transformed with pREP41-FLAG was used as control. Native cell extracts were precipitated with anti-FLAG antibody and resolved on western blot. Membrane was cut and upper part was developed with anti-FLAG, whereas bottom was developed with anti-PCNA. Ponceau S is enclosed as loading control. (**D**) Co-immunoprecipitation of spSrs2-FLAG and endogenous Rad51. WT transformed with pREP41-FLAG was used as control. Native cell extracts were precipitated with anti-FLAG antibody and resolved on western blot. Membranes were cut and upper part was developed with anti-FLAG, whereas bottom was developed with anti-Rad51. Ponceau S is enclosed as loading control.

### *Schizosaccharomyces pombe* Srs2 counteracts spontaneous accumulation of HR factors in the nucleus as its *S. cerevisiae* counterpart but uniquely promotes accumulation of Rad51 foci when overproduced

Despite differences in the C-terminal protein structure between Srs2 orthologues from budding and fission yeast, we showed that the interactions with PCNA and Rad51 described for scSrs2 are conserved for spSrs2 in *S. pombe* (Fig. 1C and D). We also demonstrated by live-cell imaging that both pREP41-scSrs2-GFP and pREP41-spSrs2-GFP formed distinct foci in the nucleus when overproduced in fission yeast cells (Supplementary Fig. S1C). Moreover, heterologous expression of the *scSRS2* gene in *S. pombe srs2*Δ mutant complemented its sensitivity to HU and CPT to the same level as expression of the sp*srs2^+^* gene. Overproduction of spSrs2-CΔ also rescued *srs2*Δ mutant sensitivity to WT levels (Fig. 2A and Supplementary Fig. S1D). Srs2 protein levels in the studied transformants were comparable (Supplementary Fig. S1E and F).

**Figure 2.**
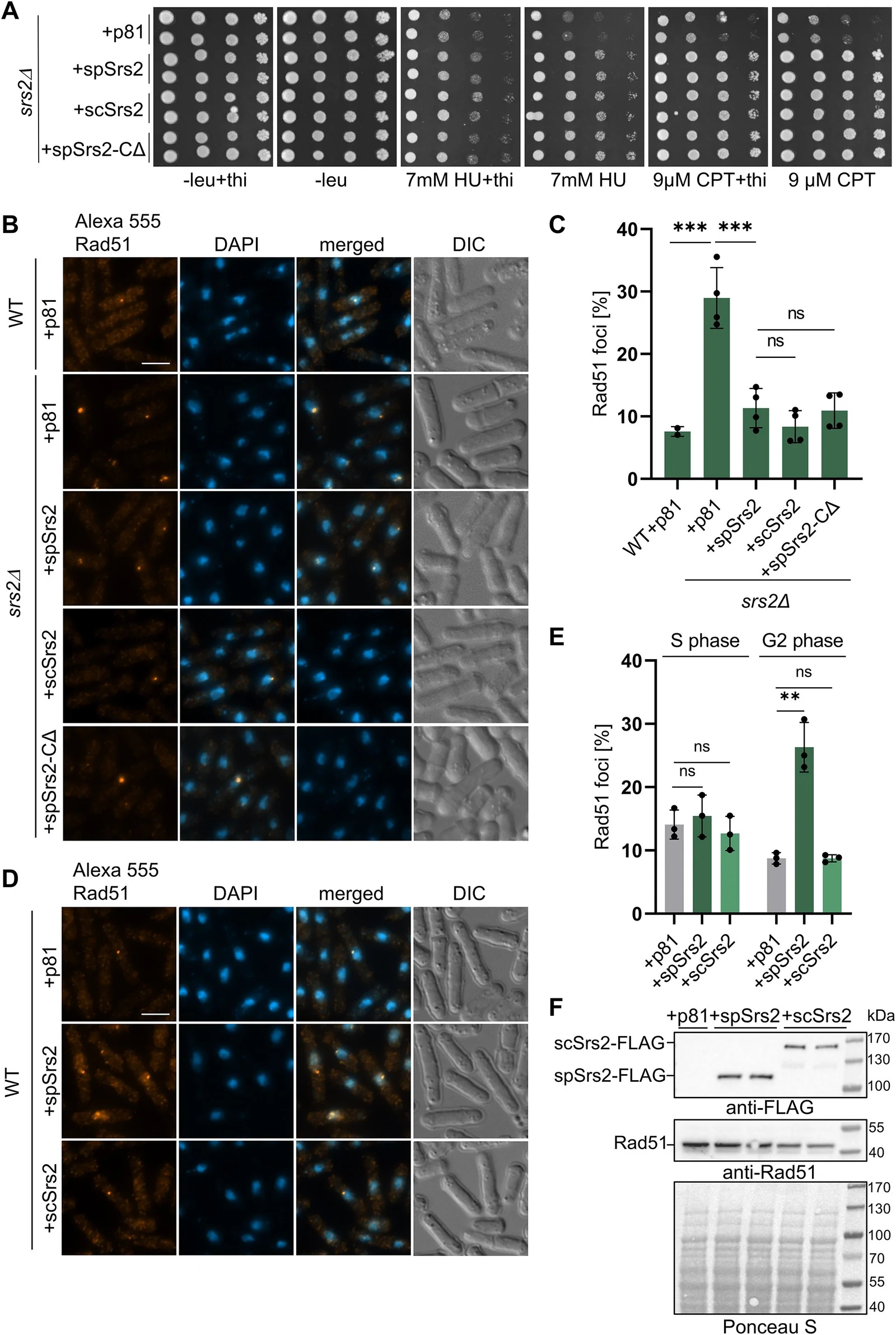
Complementation of the *srs2^+^* deletion by spSrs2, scSrs2, and spSrs2-CΔ. (**A**) Drop dilution assay of *srs2*Δ strain transformed with pREP81-FLAG, pREP81-spSrs2-FLAG, pREP81-scSRS2-FLAG, or pREP81-spSrs2-CΔ(1–877)-FLAG plasmids on indicated drugs. Cells were appropriately diluted and spotted on EMM plates containing HU or CPT, with or without thiamine (to suppress or induce plasmid expression, respectively). (**B**) Representative immunofluorescence images of endogenous Rad51 protein in indicated transformants. Scale bar = 5 μm. (**C**) Percentage of cells forming Rad51 foci in indicated transformants. At least 400 cells were analysed for each transformant. Dots represent values for independent biological replicates. Error bars show the SD around the mean values. Two-sided Student’s *t-*test was used to calculate the *P-*values. (**D**) Representative immunofluorescence images of endogenous Rad51 protein in indicated transformants. Scale bar = 5 μm. (**E**) Percentage of cells forming Rad51 foci in indicated transformants. At least 400 cells were analysed for each transformant. Dots represent values for independent biological replicates. Error bars show the SD around the mean values. Two-sided Student’s *t-*test was used to calculate the *P-*values. (**F**) Western blot of total cell extracts presenting levels of scSrs2-FLAG, spSrs2-FLAG, and Rad51 in analysed transformants. Membrane was cut, the upper part was developed with anti-FLAG and lower part with anti-Rad51. Ponceau S is included as loading control.

*Saccharomyces cerevisiae* Srs2 has a well-established role in the removal of Rad51 recombinase from DNA [44–46], and accordingly we found using immunofluorescence that the number of endogenous Rad51 foci greatly increases in cells of the *S. pombe srs2*Δ mutant (Fig. 2B and C; Supplementary Fig. S2A and B). Accumulation of Rad51 on DNA in these cells is apparent in S-phase but is even greater in G2 (Supplementary Fig. S2A and B), suggesting that DNA replication is perturbed in this mutant by excessive accumulation of Rad51. These problems are not resolved before completion of bulk DNA synthesis, possibly leading to DNA damage and further Rad51 loading on DNA in G2. Overproduction of scSrs2 or spSrs2 as well as spSrs2-CΔ in *srs2Δ* cells complemented this phenotype as evidenced by the decrease in the number of spontaneous Rad51 foci to the WT level (Fig. 2B and C). However, as the spSrs2-CΔ variant retains the ability to bind PCNA, we cannot assess the importance of this interaction for Rad51 regulation. Taken together, these data underscore similarities between Srs2 function in the regulation of Rad51 in both yeasts.

The ability of the *S. cerevisiae* Srs2 helicase to remove not only Rad51 recombinase but also Rad52 mediator and single-stranded binding protein, RPA, from DNA is well documented [16, 47]. Similarly in *S. pombe* we observed in the *srs2*Δ mutant an increase in the number of RPA foci (by visualizing Rad11-GFP, a large subunit of the RPA complex) (Supplementary Fig. S2C and D) and Rad52-GFP foci (Supplementary Fig. S2E and F). This indicates that *S. pombe* Srs2, as its *S. cerevisiae* counterpart, is important for the regulation of Rad52 and RPA protein levels on DNA.

Overproduction of scSrs2 leads to a mild growth defect in WT *S. cerevisiae*, and its toxicity increases in HR-deficient mutants [48]. We have recently shown that also in *S. pombe* the upregulation of spSrs2 levels results in an increased rate of chromosome loss, checkpoint activation, and a slight loss of viability [35]. We thus examined the accumulation of Rad51 on DNA in cells overproducing spSrs2 and somewhat surprisingly found more Rad51 foci in their nuclei. Moreover, contrary to what we observed in *srs2*Δ mutant, accumulation of Rad51 in spSrs2-overproducing cells was apparent only in G2 phase of the cell cycle (Fig. 2D and E). This suggests that Rad51 loading during replication is not perturbed. It is possible that upregulation of spSrs2 levels leads to unscheduled removal of Rad51 from DNA, possibly resulting in DNA damage and subsequent increased accumulation of Rad51 in G2 cells. Alternatively, DNA unwinding by spSrs2 may expose ssDNA and create a substrate for Rad51 binding, leading to its increased accumulation in G2. Accordingly, an increased number of bright RPA foci was previously observed in spSrs2-overproducing cells [35], indicating that the excess of ssDNA is formed. A distant human Srs2 orthologue, WRN RecQ ATPase, has been shown to collaborate with DNA2 in the processing of stalled replication intermediates to generate 3′ ssDNA overhangs and enable Rad51-dependent replication restart [49]. Interestingly, *S. pombe* Dna2 was also shown to cleave DNA strands of a model replication fork, but the involvement of Srs2 or other helicases was not assessed [50]. Hence, the role of spSrs2 in modulating Rad51 occupancy on DNA is complex, as not only *srs2^+^* depletion, but also its overexpression, result in Rad51 accumulation, and moreover, the underlying cause is most probably different. It is worth noting that the accumulation of Rad51 was specific only for cells overproducing spSrs2, because heterologous expression of the sc*SRS2* gene had no effect on the number of Rad51 foci in fission yeast cells (Fig. 2D and E) even though the levels of both proteins in the studied transformants were comparable (Fig. 2F). Thus, some potentially toxic activity of spSrs2 that results in the accumulation of Rad51 on DNA is not shared by its *S. cerevisiae* counterpart, at least when it is expressed in *S. pombe* cells.

### Interaction of spSrs2 with the Rrp1 translocase

As described in the introduction, we have previously shown that fission yeast Srs2 is involved in a HR pathway together with Rrp1–Rrp2 complex [22] and subsequently demonstrated that Rrp1 is a translocase that interacts with Rad51 and regulates its binding to dsDNA [13]. Subsequently, we found that Rrp1 localized to the centromeres, while spSrs2 was present at the rDNA locus [35, 51]. Here we show that spSrs2 co-immunoprecipitates with Rrp1 (Fig. 3A) and is also enriched at the centromeres, as demonstrated using ChIP (Fig. 3B). Moreover, we showed by ChIP that Rrp1 interacts not only with centromeres as previously reported but is also enriched at the rDNA locus (Fig. 3C). We confirmed these ChIP data by live-cell imaging demonstrating colocalization of overproduced spSrs2-GFP or Rrp1-GFP with inner kinetochore protein Mis6-RFP (Supplementary Fig. S3A) and with rDNA region visualized by Gar2-mCherry (Supplementary Fig. S3B). Together, these results suggested that since both proteins interact and are present at similar genomic loci, they may jointly regulate Rad51 activity.

**Figure 3.**
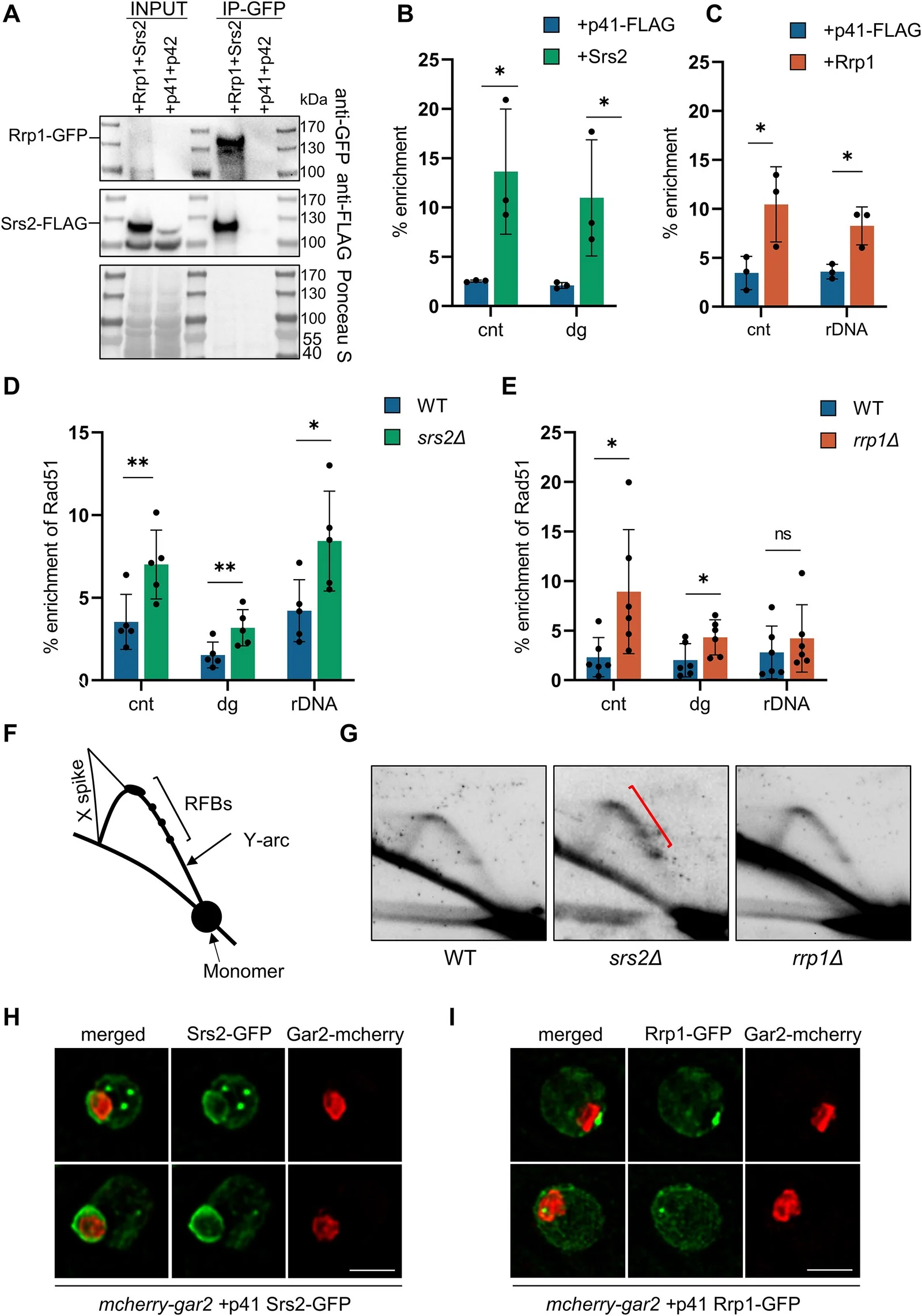
Fission yeast Srs2 (referred in the text as spSrs2) interacts with Rrp1 and binds to similar genomic sites. (**A**) Co-immunoprecipitation of Rrp1-GFP and Srs2-FLAG expressed from pREP41 and pREP42, respectively. WT transformed with pREP42 and pREP41-GFP was used as control. Native cell extracts were precipitated with anti-GFP antibody and resolved on western blot. Membranes were developed with anti-GFP and anti-FLAG. Ponceau S is enclosed as loading control. (**B**) ChIP of Srs2-FLAG expressed from pREP41 at indicated loci. Normalized values presented as % of enrichment. Intergenic locus was used as an internal specificity control. Dots show independent biological replicates. *P < .05; cnt – core of centromere, dg – outer repetitive sequences of centromere. Error bars: SD around the mean values. Two-sided Student’s *t-*test was used to calculate the *P-*values. (**C**) ChIP of Rrp1-FLAG expressed from pREP41 at centromere and rDNA. Normalized values presented as % of enrichment. Intergenic locus was used as an internal specificity control. Dots show independent biological replicates. *P < .05; cnt – core of centromere. Error bars: SD around the mean values. Two-sided Student’s *t-*test was used to calculate the *P-*values (**D, E**) ChIP of endogenous Rad51 at centromeres and rDNA in *srs2Δ* and *rrp1Δ* mutants, respectively. Normalized values presented as % of enrichment. Intergenic locus was used as an internal specificity control. Dots show independent biological replicates. *P < .05 cnt – core of centromere, dg – outer repetitive sequences of centromere. Error bars: SD around the mean values. Two-sided Student’s *t-*test was used to calculate the *P-*values. (**F**) Diagram of the replication profile (Y-arc) obtained by 2D gel electrophoresis of the rDNA locus. RFBs – replication fork barriers. Monomer – 3.1 kb BamHI fragment derived from genomic DNA. (**G**) Representative pictures of Southern blot analysis of rDNA replication in WT cells, *srs2Δ*, and *rrp1Δ* mutants. The red bracket indicates increased stalling of replication forks in *srs2Δ* at programmed RFBs in rDNA. Data from three independent experiments are presented here and in Supplementary Fig. S3D. (**H, I**) High-resolution microscopy images of co-localization between Srs2-GFP or Rrp1-GFP with rDNA marker Gar2-mCherry, respectively. Scale bar = 2 μm.

We thus examined by ChIP the binding of Rad51 to centromeres and rDNA in *srs2*Δ and *rrp1*Δ mutants. We found that in cells lacking *srs2^+^*, Rad51 accumulated at rDNA and within core and outer repeats of centromeres (Fig. 3D). Rrp1 has been previously shown to regulate Rad51 DNA binding specifically at the centromeres [13], and here we recapitulate this result, but interestingly, we also show that Rrp1, in contrast to spSrs2, does not influence Rad51 occupancy at the rDNA region (Fig. 3E). We have previously shown using bidimensional gel electrophoresis that depletion of spSrs2 leads to increased replication fork stalling within the rDNA region [35]. Here we confirm that in the *srs2*Δ mutant, replication arrests more frequently at programmed RFBs present in this region, while Rrp1 depletion has no pronounced effect (Fig. 3F and G; Supplementary Fig. S3C and D).

These results indicate that Rrp1, as opposed to spSrs2, is not involved in regulation of Rad51 within the rDNA region and is not involved in the replication of rDNA arrays which is surprising given the localization of overproduced Rrp1 in the nucleolus. However, when we examined by super-resolution microscopy the localization of spSrs2-GFP and Rrp1-GFP to the rDNA region, visualized by Gar2-mCherry staining, we found that the nature of interaction with rDNA is different for both proteins. spSrs2-GFP was present as a layer on the entire outside surface of the nucleolus, with additional distinct foci clearly separate from this structure (Fig. 3H), whereas Rrp1-GFP formed defined foci, some of which were adjacent to or occasionally co-localized with the rDNA (Fig. 3I). This may account for the differential involvement of spSrs2 and Rrp1 in this region.

### Rrp1 does not target Rad51 accumulating on DNA when spSrs2 is absent or overproduced

We have shown above that heterologous expression of scSrs2 in fission yeast *srs2*Δ mutant is able to complement both its genotoxin sensitivity and excessive accumulation of Rad51 on DNA (Fig. 2A–C). Because overproduction of Rrp1, Fbh1, or spSrs2 has been recently found to reduce Rad51 accumulation in *rad54Δ* cells [31], we decided to check if overexpression of *rrp1^+^*might alleviate the phenotypes of *srs2Δ* mutant. We found that overproduced Rrp1 is unable to suppress the sensitivity of *srs2*Δ to HU and CPT (Fig. 4A). Additionally, Rrp1 cannot remove Rad51 accumulating in cells of the *srs2*Δ mutant, either in S or G2 phase of the cell cycle (Fig. 4B and C). The expression of the *rrp1^+^* gene in analysed transformants was confirmed by western blot, and the level of Rad51 was unaffected in indicated strains (Fig. 4D).

**Figure 4.**
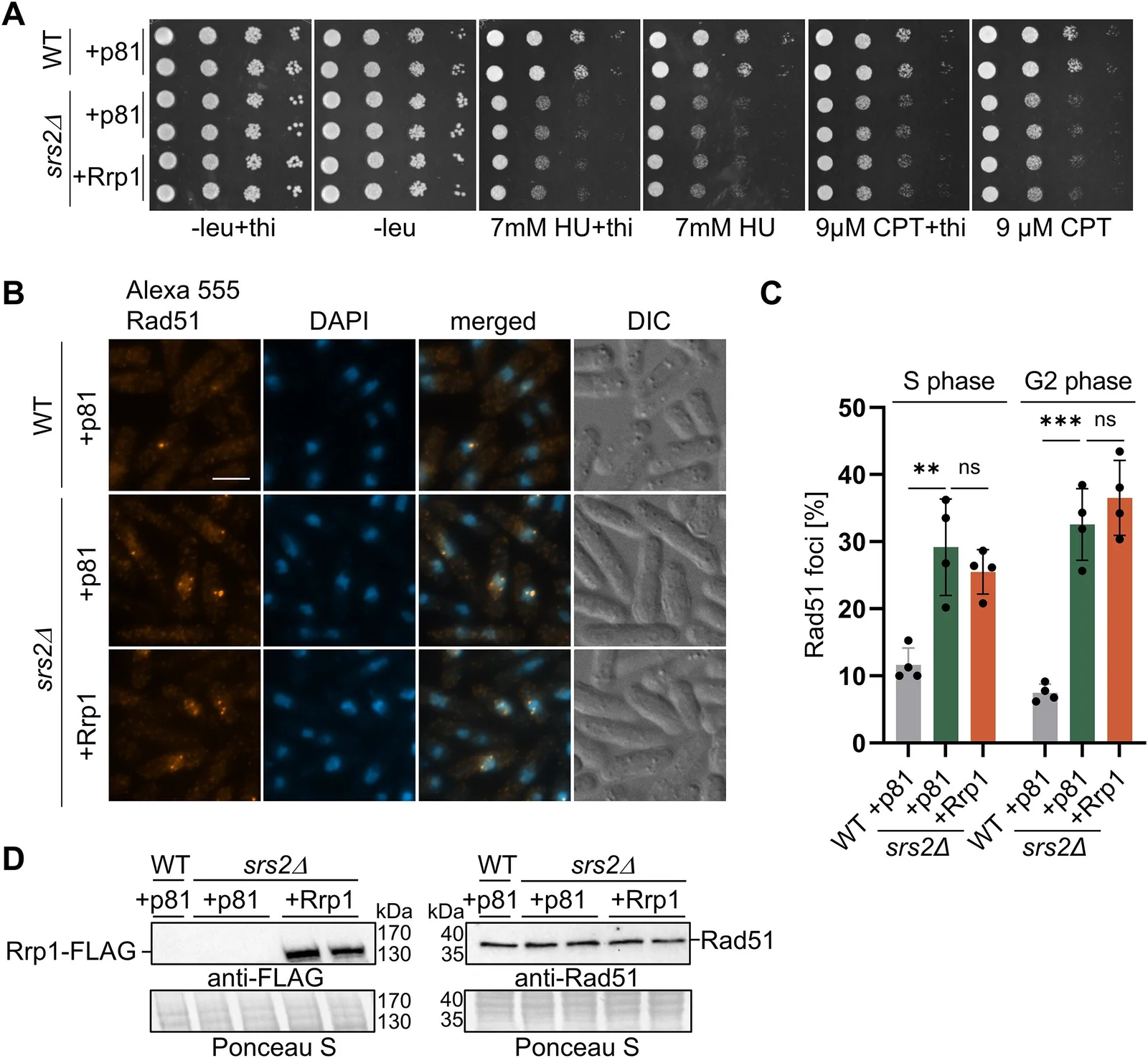
Rad51 foci arising in the absence or excess of *S. pombe* Srs2 are not removed by Rrp1. (**A**) Drop dilution assay of *srs2*Δ strain transformed with empty pREP81-FLAG or pREP81-Rrp1-FLAG plasmids on indicated drugs. WT transformed with empty pREP81-FLAG plasmid was used as control. Cells were appropriately diluted and spotted on EMM plates containing HU or CPT, with or without thiamine (to suppress or induce plasmid expression, respectively). (**B**) Representative immunofluorescence images of endogenous Rad51 protein in indicated transformants. Scale bar = 5 μm (**C**) Percentage of cells forming Rad51 foci in indicated transformants in S and G2 phases, respectively. At least 500 cells were analysed for each transformant. Dots represent values for independent biological replicates. Error bars show the SD around the mean values. Two-sided Student’s *t-*test was used to calculate the *P-*values. (**D**) Western blot of total cell extracts presenting levels of Rrp1-FLAG and endogenous Rad51 in analysed transformants. Ponceau S is included as loading control.

In WT cells, overproduction of spSrs2, Rrp1, or both proteins together results in comparable levels of replication stress, observed as an increase in the number of cells with EdU signal in G2 phase, which showed delayed replication (Fig. 5A and B). Additionally, we examined the frequency of co-localization between Rad11-GFP and Rad51 foci by double immunofluorescence in transformants overproducing spSrs2 or Rrp1. Consistent with the data for EdU incorporation, suggesting enhanced replication stress, overexpression of *srs2^+^, rrp1^+^*, or both led to an increase in Rad11-GFP foci, indicating that excessive levels of ssDNA accumulated in these cells (Fig. 5C and D). Yet upregulation of spSrs2 and Rrp1 has opposite effects on the number of Rad51 foci in G2 cells, leading to their increase and decrease, respectively (Supplementary Fig. S3E and F). Moreover, in transformants co-expressing *rrp1^+^* and *srs2^+^*, no marked decrease of the elevated number of Rad51 foci was observed (Supplementary Fig. S3E and F; Fig. 5C–E). This indicates that Rrp1 cannot counteract the excessive accumulation of Rad51 in cells overproducing spSrs2, as it seems to target only a subfraction of Rad51 molecules that do not colocalize with Rad11, so presumably are bound to dsDNA (Fig. 5C–F). Our data suggest that Rrp1 and spSrs2 seem to engage with specific pools of Rad51: Rrp1 may be active predominantly within the centromeres, while spSrs2 could regulate Rad51 also in other regions of the genome, such as rDNA (Fig. 3). However, when the levels of respective proteins are upregulated, their genomic occupancy may change, and indeed we observe more prominent replication fork stalling in cells overexpressing *rrp1^+^*when replication profiles were analysed by bidimensional gel electrophoresis, while *srs2^+^*overproduction seemed to have a WT profile, in contrast to mutant devoid of *srs2^+^* (Fig. 5G and Supplementary Fig. S4A).

**Figure 5.**
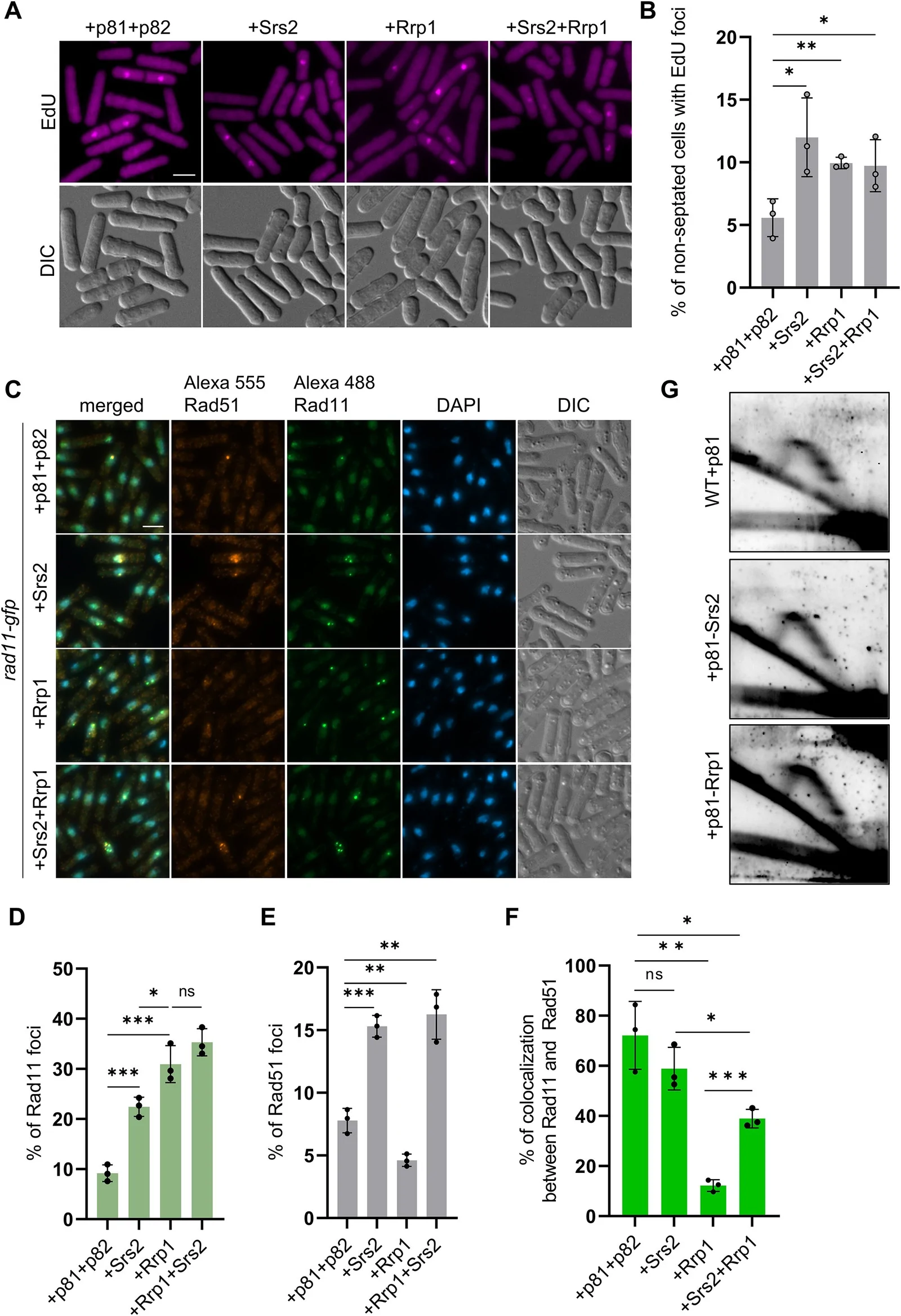
Overproduction of fission yeast Srs2 and Rrp1 leads to increased replication stress and ssDNA formation but differently affect Rad51 binding to DNA. (**A**) Example of images of EdU incorporation in logarithmic, septated (S-phase) cells and non-septated (G2 phase) in indicated transformants (strains transformed with pairs of plasmids: pREP82-HA and pREP81-FLAG, pREP82-HA and pREP81-Srs2-FLAG, pREP82-HA-Rrp1 and pREP81-FLAG, pREP82-HA-Rrp1 and pREP81-Srs2-FLAG). Scale bar = 5 μm. (**B**) Quantification of non-septated cells forming EdU foci in G2 phase, marking delayed DNA replication, occurring outside S-phase in indicated strains (shown as %). Dots represent values obtained from independent biological experiments. At least 600 cells were analysed for each strain for a single experiment. Error bars show the SD around the mean values. Two-sided Student’s *t-*test was used to calculate the *P-*values. (**C**) Representative double-immunofluorescence images of endogenous Rad51 and tagged Rad11-GFP protein in indicated transformants (strains transformed with pairs of plasmids: pREP82-HA and pREP81-FLAG, pREP82-HA and pREP81-Srs2-FLAG, pREP82-HA-Rrp1 and pREP81-FLAG, pREP82-HA-Rrp1 and pREP81-Srs2-FLAG). Scale bar = 5 μm. (**D**) Percentage of cells forming Rad11 foci in the indicated strains used in panel (C). (**E**) Percentage of cells with Rad51 foci in the indicated strains used in panel (C). In both (D) and (E), dots indicate independent biological replicates. At least 400 cells were analysed. Error bars show the SD around the mean values. Two-sided Student’s *t-*test was used to calculate the *P-*values. (**F**) Percentage of colocalization between Rad11 and Rad51 in the indicated strains. Cells containing Rad11 foci were used as the total population for calculating colocalization with Rad51 foci. Dots indicate independent biological replicates. At least 400 cells were analysed. Error bars show the SD around the mean values. Two-sided Student’s *t-*test was used to calculate the *P-*values. Data in panels (D) and (E) were obtained from the same experiment and represent analyses of the same cells. (**G**) Southern blot analysis of rDNA replication by bidimensional gel electrophoresis in WT cells transformed with pREP81-Srs2-FLAG, pREP81-Rrp1-FLAG, or empty pREP81-FLAG as control. Data from three independent experiments are presented here and in Supplementary Fig. S4A.

Rrp1, as a dsDNA translocase, may predominantly affect not only Rad51, as has been demonstrated in [13], but also other proteins bound to dsDNA rather than ssDNA. Consistent with this notion, we show that Rrp1 did not counteract the spontaneous accumulation of either RPA or Rad52 in the nucleus, since no increase in the number of Rad11-GFP (Supplementary Fig. S4B and C) or Rad52-GFP foci (Supplementary Fig. S4D and E) was detected in the *rrp1*Δ mutant. Additionally, overproduction of Rrp1 did not lead to the removal of RPA (Supplementary Fig. S4F and G) and Rad52 (Supplementary Fig. S4H and I) bound to DNA in *srs2*Δ mutant but rather resulted in an increase in the number of their respective foci.

### Upregulation of spSrs2 levels does not alleviate the toxicity of Rad51 overproduction and counteracts the effect of the Rrp1 DNA translocase

Excessive Rad51 binding to long stretches of dsDNA is toxic in human cells [12, 52]. We have previously shown in *S. pombe* a severe growth defect of a strain where GFP-Rad51 was overproduced. This was further exacerbated by deletion of *rrp1^+^*, while overexpression of *rrp1^+^* suppressed the loss of viability induced by the excess of Rad51 binding [13].

Here, we corroborated these results in two experimental setups: with untagged *rad51^+^* expressed from a plasmid under the control of medium-strength *nmt41* promoter (Supplementary Fig. S5A) and with a strain with integrated *GFP-rad51^+^*gene expressed from strong *nmtP3* promoter (Fig. 6A). Somewhat surprisingly, however, we found that in both systems overexpression of *srs2^+^* does not alleviate the toxicity of Rad51 overproduction (Supplementary Fig. S5A and Fig. 6A). We thus examined the studied transformants in the GFP-Rad51-overproducing strain by growth curve analysis and confirmed that while upregulation of Rrp1 levels resulted in the rescue of its growth defect, *srs2^+^* overexpression did not (Fig. 6B, green and orange lines, respectively). Moreover, when both Rrp1 and spSrs2 were overproduced, the rescue conferred by Rrp1 was partially reversed (Fig. 6B, blue and green lines, respectively). Importantly, Rad51, spSrs2, and Rrp1 protein levels in all studied transformants were comparable (Supplementary Fig. S5B and C), so the observed effect did not stem from lower amounts of Rrp1 in *srs2^+^* overexpressing cells.

**Figure 6.**
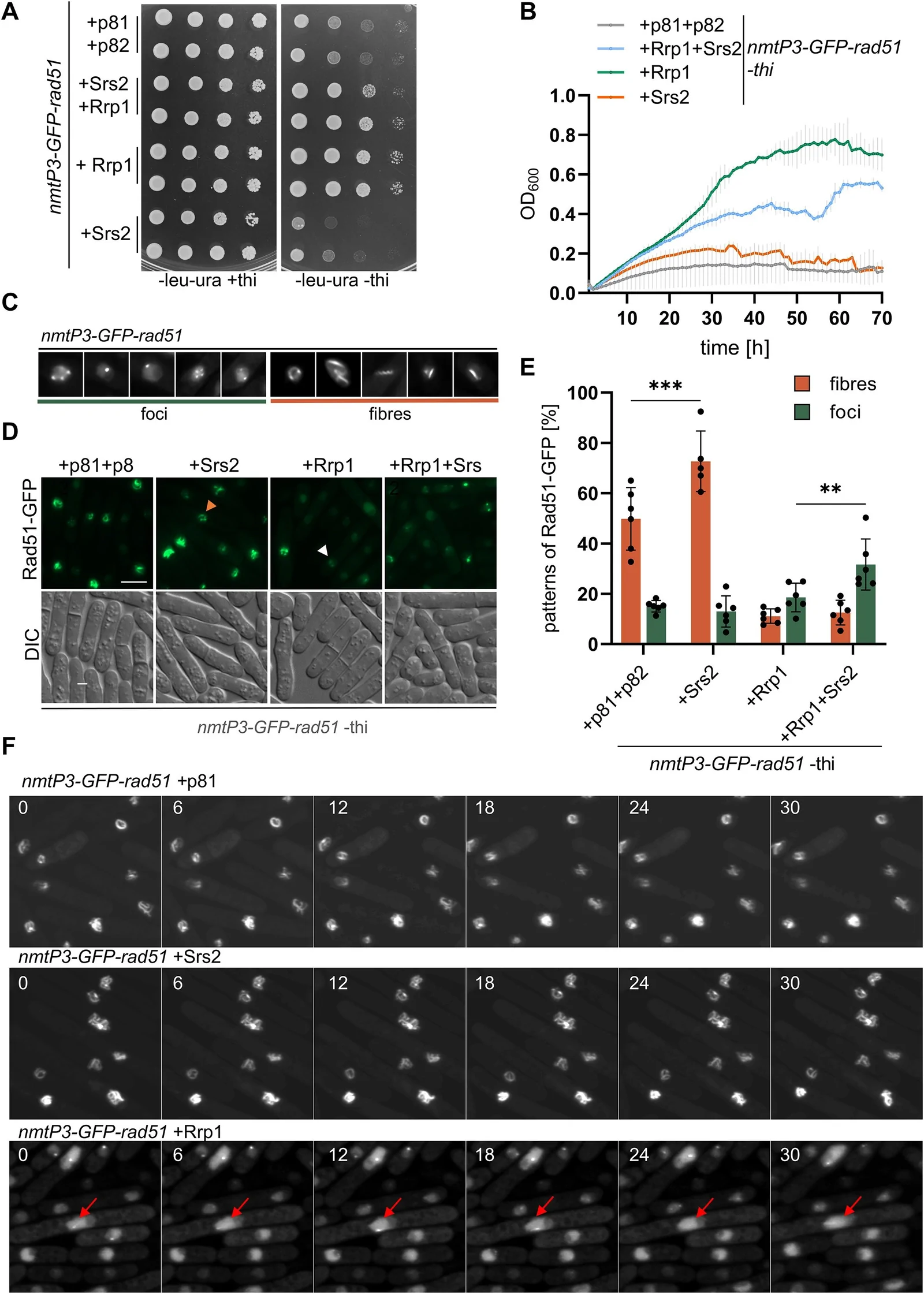
Upregulation of *S. pombe* Srs2 increases toxicity of Rad51 overproduction and counteracts Rrp1 activity. (**A**) Drop dilution assay of nmtP3-GFP*-*rad51 strain transformed with indicated pairs of plasmids (pREP82-HA and pREP81-FLAG, pREP82-HA and pREP81-Srs2-FLAG, pREP82-HA-Rrp1 and pREP81-FLAG, pREP82-HA-Rrp1 and pREP81-Srs2-FLAG) under repressive (+thi) or induced (-thi) conditions. (**B**) Growth curves of indicated transformants under conditions without thiamine. (**C**) Examples of foci and fibres formed by overexpressed GFP-Rad51. (**D**) Representative images of GFP*-*Rad51 structures in indicated transformants upon induction of expression (-thi). Scale bar = 5 μm (**E**) Percentage of overproduced GFP*-*Rad51 patterns in indicated transformants (-thi). At least 400 cells were analysed. Dots represent independent biological replicates. Error bars show the SD around the mean values. Two-sided Student’s *t-*test was used to calculate the *P*-values. (**F**) Time-lapse light sheet microscopy of strains expressing nmtP3-GFP-rad51 with empty vector (+p81), Srs2 or Rrp1. Montage of 30 min movies shows frames in 6 min intervals across acquisition time.

Upregulation of Rad51 leads to the formation of genotoxic Rad51 protein complexes on undamaged chromatin, leading to viability loss. In human cells, factors like FIGNL1 safeguard genome stability by preventing the formation of excessive Rad51 aggregates on DNA and replication fork collapse as well as suppressing ultra-fine bridge formation, which is overall crucial for cell survival [53]. We have previously demonstrated in fission yeast that overexpression of *rrp1*^+^ in the *nmtP3-GFP-rad51* strain resulted in the disappearance of numerous Rad51 fibres from DNA [13], consistent with the rescue of Rad51 growth defect it provides. Here, we surprisingly found that overproduction of Srs2, as opposed to Rrp1, in cells overproducing GFP-Rad51, leads to an increase in the number of Rad51 fibres (Fig. 6C–E). Similar results were obtained when Srs2 was overproduced in cells overexpressing untagged *rad51*^+^ (Supplementary Fig. S5D and E). This suggests that Srs2 is not only unable to remove excessive amounts of Rad51 forming fibres on dsDNA, but that under *rad51^+^* overexpression conditions, upregulation of Srs2 levels leads to further accumulation of Rad51 on chromatin.

It has been shown in *S. pombe* that Fbh1, an F-box helicase, also removes Rad51 from ssDNA as well as ubiquitinates it, which is thought to diminish the ability of Rad51 to rebind to DNA. Moreover, Rad51 foci have been found to accumulate in cells devoid of Fbh1 both in *S. pombe* and in human cells [27, 33, 54, 55]. We demonstrated that the toxicity of GFP-Rad51 overproduction is slightly enhanced (Supplementary Fig. S6A) and the number of Rad51 fibres on DNA also increases in both *srs2*Δ *nmtP3-GFP-rad51* and *fbh1Δ nmtP3-GFP-rad51* mutant backgrounds (Supplementary Fig. S6B and C). When overexpressed, Fbh1 did not rescue the growth defect caused by elevated GFP-Rad51 levels, similar to spSrs2 and in contrast to Rrp1 (Supplementary Fig. S6D). Introduction of the sc*SRS2* gene into the *nmtP3-GFP-rad51* strain also did not rescue its growth defect (Supplementary Fig. S6D). Moreover, the presence of overproduced Fbh1 slightly increased the number of Rad51 fibres on DNA, in contrast to overproduction of Rrp1, but similar to spSrs2 (Supplementary Fig. S6E and F). Overproduction of scSrs2 had no effect on the number of Rad51-GFP foci or fibres (Supplementary Fig. S6E and F) underscoring the difference between these two yeast orthologues. The levels of the examined proteins were comparable (Supplementary Fig. S6G). Our data demonstrate that in the absence of *rrp1^+^*and *srs2^+^*, as well as *fbh1^+^*, the growth defect due to excessive Rad51 accumulation on DNA increased. Interestingly, however, only upregulation of Rrp1, targeting dsDNA-bound Rad51, could alleviate the toxicity of Rad51 overproduction. Overproduction of spSrs2 or Fbh1 does not suppress the detrimental effects of excessive Rad51 accumulation on undamaged chromatin, presumably because they preferentially regulate Rad51 bound to ssDNA.

To examine the effect of Rrp1 and Srs2 overproduction on Rad51 fibres in real time, we employed light-sheet microscopy. In the course of a 30-min acquisition, we did not observe any changes in the appearance of structures formed by Rad51 in *nmtP3-GFP-rad51* strain transformed with empty vector or *srs2^+^* gene. Only in cells expressing *rrp1^+^* gene we could detect events when Rad51 focus repeatedly expanded into a short filament and then shrunk back into a round focus (Fig. 6F). We interpret these data to mean that Rrp1 restricts Rad51 expansion on DNA beyond the initial points of nucleation, which presumably can be localized at sites of replication fork stalling and/or DNA damage.

Interestingly, upon concomitant overexpression of *srs2^+^* and *rrp1^+^* in *GFP-Rad51* overproducing cells, a greater number of GFP-Rad51 foci could be observed than in *rrp1^+^* and *GFP-rad51^+^* overexpressing control (Fig. 6D and E), even though the number of Rad51 fibres remained low. This suggests that higher levels of Rad51 remaining on chromatin are the cause of the weaker rescue of the *nmtP3-GFP-rad51* strain’s growth defect when both Rrp1 and spSrs2 are overproduced, described earlier (Fig. 6A and B), and raises the possibility that spSrs2 may directly antagonize the effect of Rrp1 activity.

The above results were obtained upon concomitant overproduction of Rad51, Rrp1, and spSrs2 as well as scSrs2 and Fbh1. We thus examined the number of GFP-Rad51 foci in relevant transformants of *nmtP3-GFP-rad51* strain cultured on medium containing thiamine, when gene expression from *nmt* promoters was repressed. Under these conditions, all proteins should be produced in the studied transformants at much lower, closer-to-native levels, and accordingly no growth defect was then observed (left panels in Fig. 6A and Supplementary Fig. S6D). Under these non-inducing conditions, no GFP-Rad51 fibres were detected in the control *nmtP3-GFP-rad51* strain, only a few foci, and while overproduction of scSrs2 had no effect, their number slightly decreased in transformants with pREP81-Rrp1-FLAG (Fig. 7A and B; Supplementary Fig. S7A and B), as described before [13]. In contrast, multiple GFP-Rad51 foci could be seen upon spSrs2 and Fbh1 overproduction (Fig. 7A and B; Supplementary Fig. S7A and B), and their number did not diminish in cells co-expressing *srs2^+^* and *rrp1+* (Fig. 7A and B). This again demonstrates that overproduction of spSrs2, but not scSrs2, promotes excessive Rad51 binding to DNA and that Rrp1 does not affect these Rad51 species, corroborating our results obtained for native Rad51 levels shown in Fig. 5C–E.

**Figure 7.**
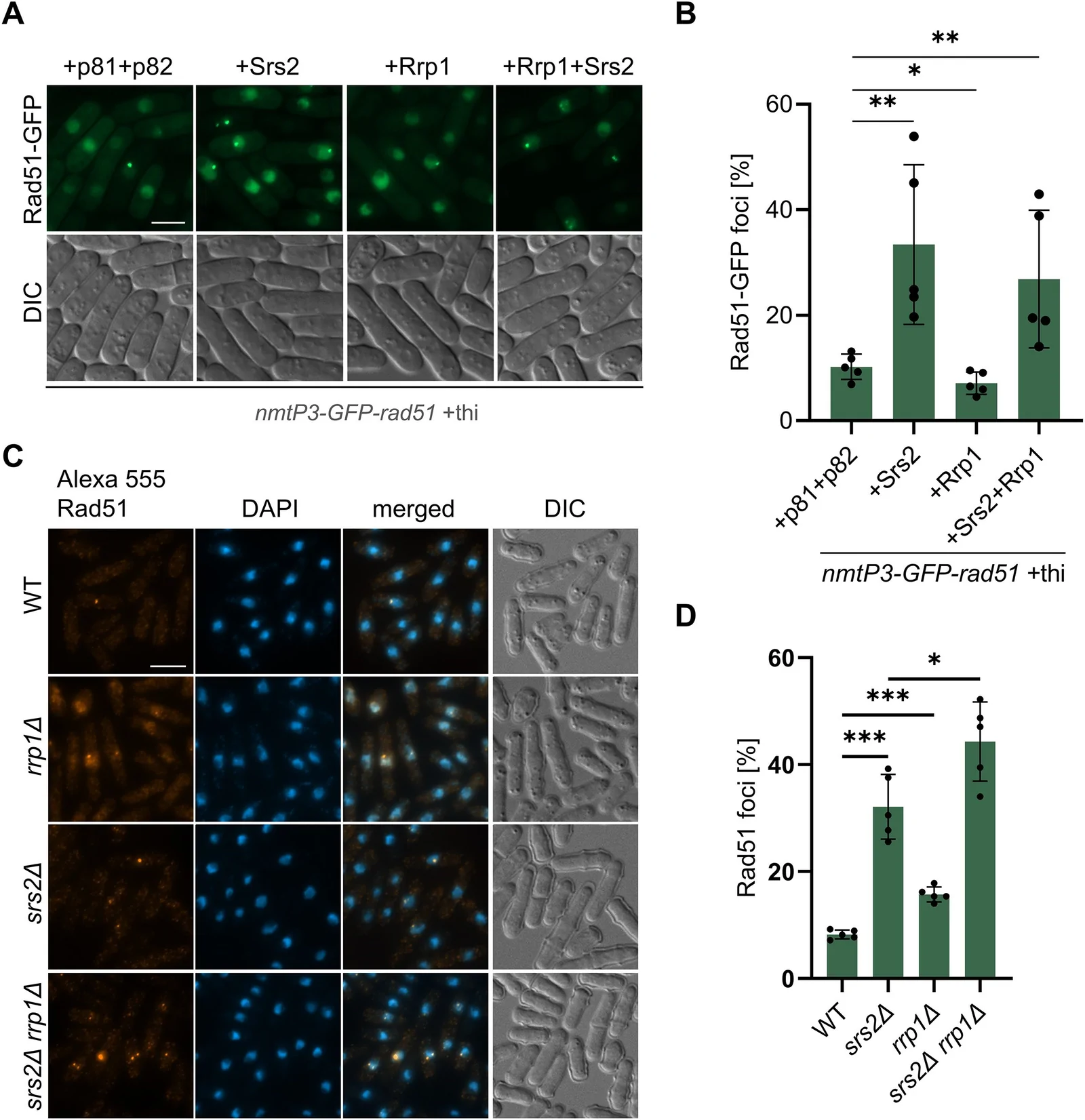
Srs2 and Rrp1 target different Rad51 pools. (**A**) Representative images of GFP*-*Rad51 structures in indicated transformants in repressive conditions (+thi). Scale bar = 5 μm. (**B**) Percentage of GFP*-*Rad51 foci formation upon repressive conditions (+thi) in indicated transformants. At least 400 cells were analysed. Dots represent independent biological replicates. Error bars show the SD around the mean values. Two-sided Student’s *t-*test was used to calculate the *P-*values. (**C**) Representative immunofluorescence images of endogenous Rad51 protein in indicated strains. Scale bar = 5 μm. (**D**) Percentage of cells forming Rad51 foci in indicated strains. At least 500 cells were analysed for each strain. Dots represent values for independent biological replicates. Error bars show the SD around the mean values. Two-sided Student’s *t-*test was used to calculate the *P*-values.

Even a small increase in Srs2 or Rrp1, under non-inducing conditions (+thi), affected endogenous Rad51. In strains transformed with pREP81-Srs2, the number of endogenous Rad51 foci increased. On the other hand, strains harbouring pREP81-Rrp1 showed a reduced number of Rad51 foci (Supplementary Fig. S7C and D).

In summary, our data suggest that spSrs2, in contrast to Rrp1, is unable to remove Rad51 bound to dsDNA in the form of long fibres, and to rescue the growth defect resulting from excessive accumulation of Rad51 recombinase on DNA. On the other hand, Rrp1 is unable to target Rad51 accumulating on DNA when spSrs2 is overproduced, as well as when it is absent. Moreover, in the double *srs2*Δ *rrp1*Δ mutant the number of Rad51 foci on DNA further increases relative to what is observed in the respective single mutants (Fig. 7C and D). This seems to argue against direct inhibition of Rrp1 activity by spSrs2, but rather for the specificity of both proteins towards different types of Rad51 molecules, located at different chromatin regions, as shown above (Fig. 3), or bound to different DNA species, for example dsDNA versus ssDNA.

### The nature of Rad51 foci targeted by Srs2 and Rrp1

In both yeasts, Rad52, a main HR mediator, facilitates Rad51 loading on DNA coated with the ssDNA-binding protein RPA [56]. We thus asked whether the excessive accumulation of spontaneous Rad51 foci observed in the *srs2*Δ mutant was dependent on the presence of Rad52. To test this, we obtained a viable *srs2*Δ *rad52*Δ strain and showed that indeed, the number of Rad51 foci was greatly diminished and comparable in both the single *rad52*Δ and double *srs2*Δ *rad52*Δ mutants (Fig. 8A and B).

**Figure 8.**
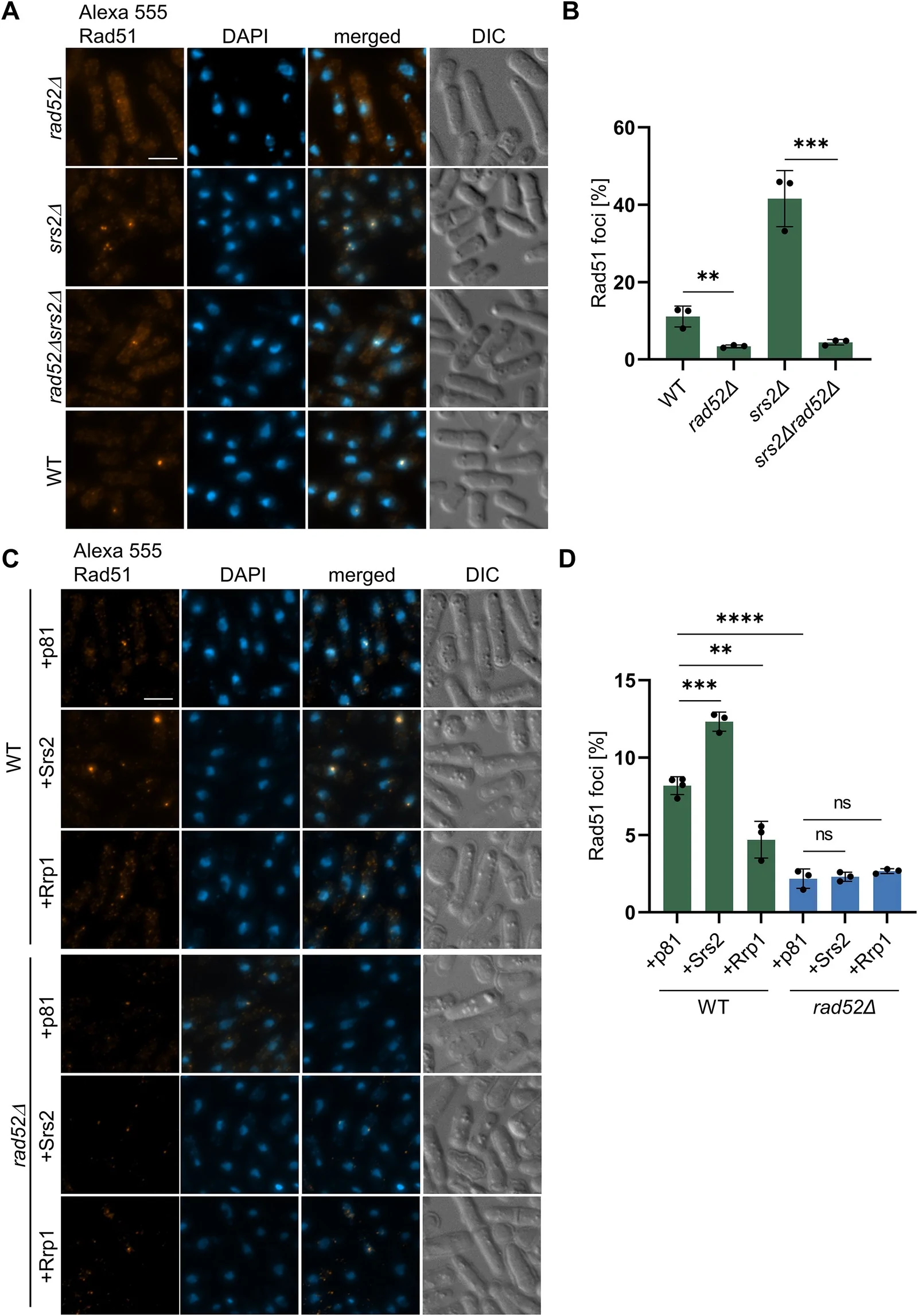
Fission yeast Srs2 promotes nucleation of Rad51 in a manner dependent on Rad52. (**A**) Representative immunofluorescence images of endogenous Rad51 protein in indicated strains. Scale bar = 5 μm. (**B**) Percentage of cells forming Rad51 foci in indicated strains. At least 500 cells were analysed for each strain. Dots represent values for independent biological replicates. Error bars show the SD around the mean values. Two-sided Student’s *t-*test was used to calculate the *P*-values. (**C**) Representative immunofluorescence images of endogenous Rad51 protein in indicated transformants. Strains transformed with empty vector (pREP81-FLAG) were used as control. Scale bar = 5 μm. (**D**) Percentage of cells forming Rad51 foci in indicated transformants. At least 500 cells were analysed for each transformant. Dots represent values for independent biological replicates. Error bars show the SD around the mean values. Two-sided Student’s *t-*test was used to calculate the *P*-values.

Interestingly, the elevated Rad51 nucleation, induced by overexpression of the *srs2^+^*gene, was also fully dependent on the presence of the HR mediator Rad52 (Fig. 8C and D). It thus suggests that spontaneous Rad51 foci accumulating both upon deletion and overexpression of *srs2^+^* helicase may represent Rad51 bound to ssDNA, possibly as canonical Rad51 nucleofilaments. The number of Rad51 foci in the *rad52*Δ mutant is lower than in WT cells overexpressing *rrp1^+^* (Fig. 8C and D), presumably because some of the foci formed in WT cells represent Rad51 bound to ssDNA or located in genomic regions inaccessible to Rrp1. Overproduction of Rrp1 in *rad52*Δ mutant did not affect the number of Rad51 foci (Fig. 8C and D), lending support to the hypothesis that initial Rad51 loading on ssDNA by Rad52 is required before it engages with dsDNA and can be removed by Rrp1.

To further investigate the nature of Rad51 molecules regulated specifically by spSrs2 and Rrp1, we took advantage of the *rad51-3A* mutant, retaining the ability to bind ssDNA and protect it from degradation, but deficient in strand-exchange activity [57, 58]. When we introduced the *rad51-3A* allele into *srs2Δ* or *rrp1Δ* backgrounds, we did not observe an increase of Rad51-3A nucleation in any of the double mutants (Fig. 9A–C, respectively), indicating that strand-exchange-deficient Rad51 molecules may be refractory to removal from DNA not only by Rrp1, which we expected, but also by spSrs2.

**Figure 9.**
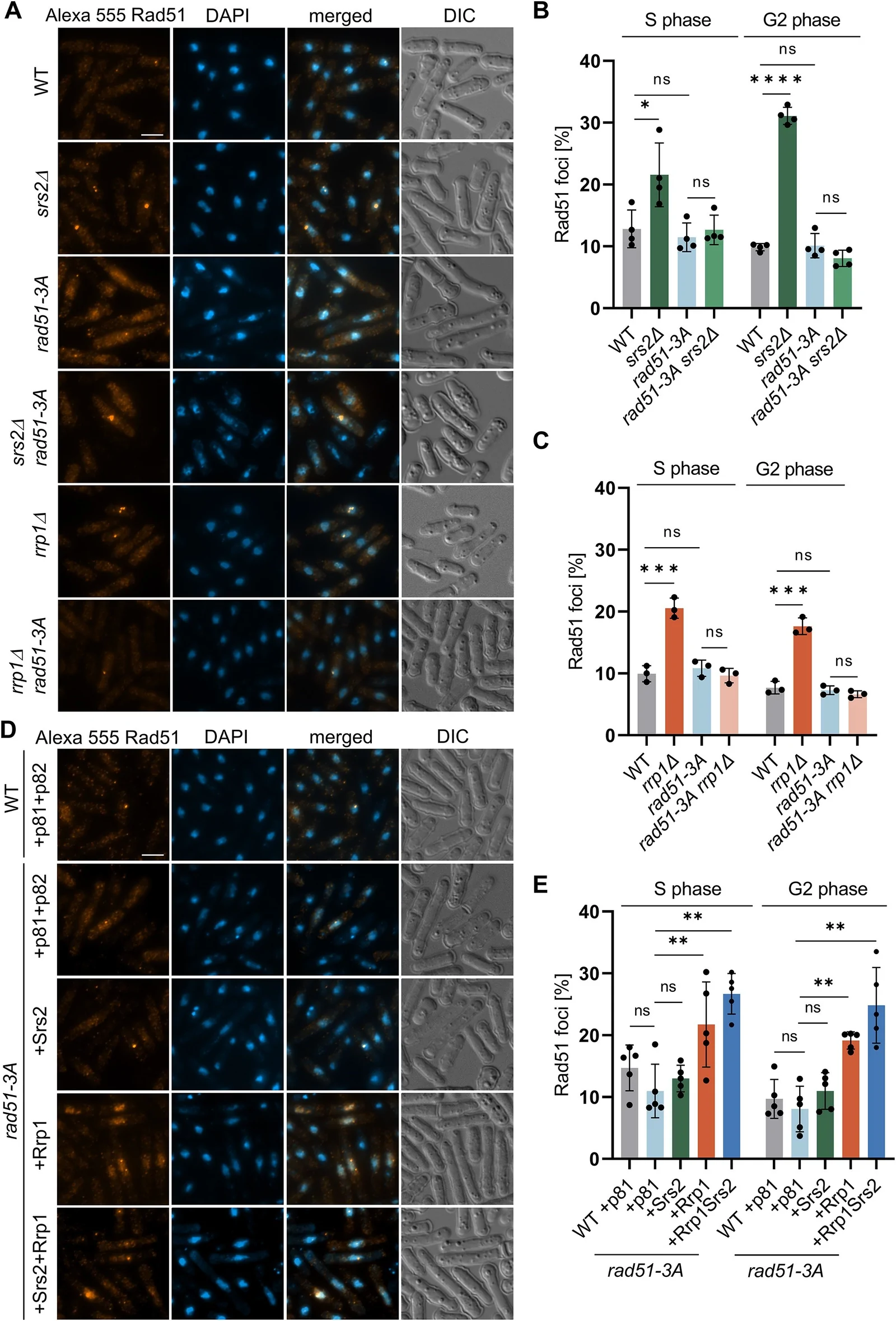
Endogenous *S. pombe* Srs2 and Rrp1 target strand-exchange-proficient Rad51, and upregulated Rrp1 might stimulate nucleation of strand-exchange-deficient Rad51-3A. (**A**) Representative immunofluorescence images of endogenous Rad51 protein in indicated strains. Scale bar = 5 μm. (**B, C**) Percentage of cells forming Rad51 foci in indicated strains. At least 400 cells were analysed for each transformant. Dots represent values for independent biological replicates. Error bars show the SD around the mean values. Two-sided Student’s *t-*test was used to calculate the *P-*values. (**D**) Representative immunofluorescence images of endogenous Rad51 protein in indicated transformants (strains transformed with pairs of plasmids: pREP82-HA and pREP81-FLAG, pREP82-HA and pREP81-Srs2-FLAG, pREP82-HA-Rrp1 and pREP81-FLAG, pREP82-HA-Rrp1 and pREP81-Srs2-FLAG). Scale bar = 5 μm. (**E**) Percentage of cells forming Rad51 foci in indicated transformants. At least 400 cells were analysed for each transformant. Dots represent values for independent biological replicates. Error bars show the SD around the mean values. Two-sided Student’s *t-*test was used to calculate the *P-*values.

Thus, upregulation of spSrs2 levels in *rad51-3A* mutant should not lead to unscheduled removal of Rad51-3A from DNA and generation of DNA damage followed by additional loading of Rad51-3A in G2 phase of the cell cycle, as we hypothesized earlier to account for the increase in Rad51 foci seen in WT cells overexpressing *srs2+* (Fig. 2D and E). Indeed, we found that overproduction of spSrs2 no longer induced enhanced Rad51-3A nucleation in this mutant (Fig. 9D and E), reinforcing the notion that spSrs2 might only target Rad51 nucleofilaments actively engaged in strand exchange.

In *S. pombe*, two protein complexes Rad55–Rad57 and Swi5–Sfr1, facilitate Rad51-mediated strand-exchange reaction, and mutations within their genes result in sensitivity to genotoxic agents and defects in HR [59]. It has been demonstrated *in vitro* that Rad52 alone cannot activate the Rad51 presynaptic filament and the presence of Swi5–Sfr1 is essential for strand exchange to occur [60, 61]. There have been no similar studies done for the spRad55–Rad57 complex, but since the *rad57*Δ *sfr1*Δ double mutant resembles *rad51Δ* [62], one can assume its role in stimulating strand exchange is also critical. To confirm our findings in a different experimental setup, we decided to examine the role of Rrp1 and Srs2 in Rad51 regulation in mutants devoid of Rad57 and Sfr1. We thus constructed a series of multiple mutants and found, as expected, that the number of Rad51 foci decreased in the nuclei of *rad57*Δ *sfr1*Δ (Fig. 10A and B), as well as *rad57*Δ and *sfr1*Δ mutants (Supplementary Fig. S8A and B), but not to the level seen in cells devoid of Rad52 (Fig. 8A and B), which is required for Rad51 loading on DNA. Importantly, additional deletion of *rrp1^+^*or *srs2^+^*in the background of these single or double mutants had no effect on the number of Rad51 foci (Fig. 10A and B; Supplementary Fig. S8A and B). Since the strand-exchange activity of Rad51 present on DNA in these mutant strains is compromised, this agrees with our results for the Rad51-3A mutant and suggests that Rrp1 and Srs2 may preferentially affect activated Rad51 nucleofilaments.

**Figure 10.**
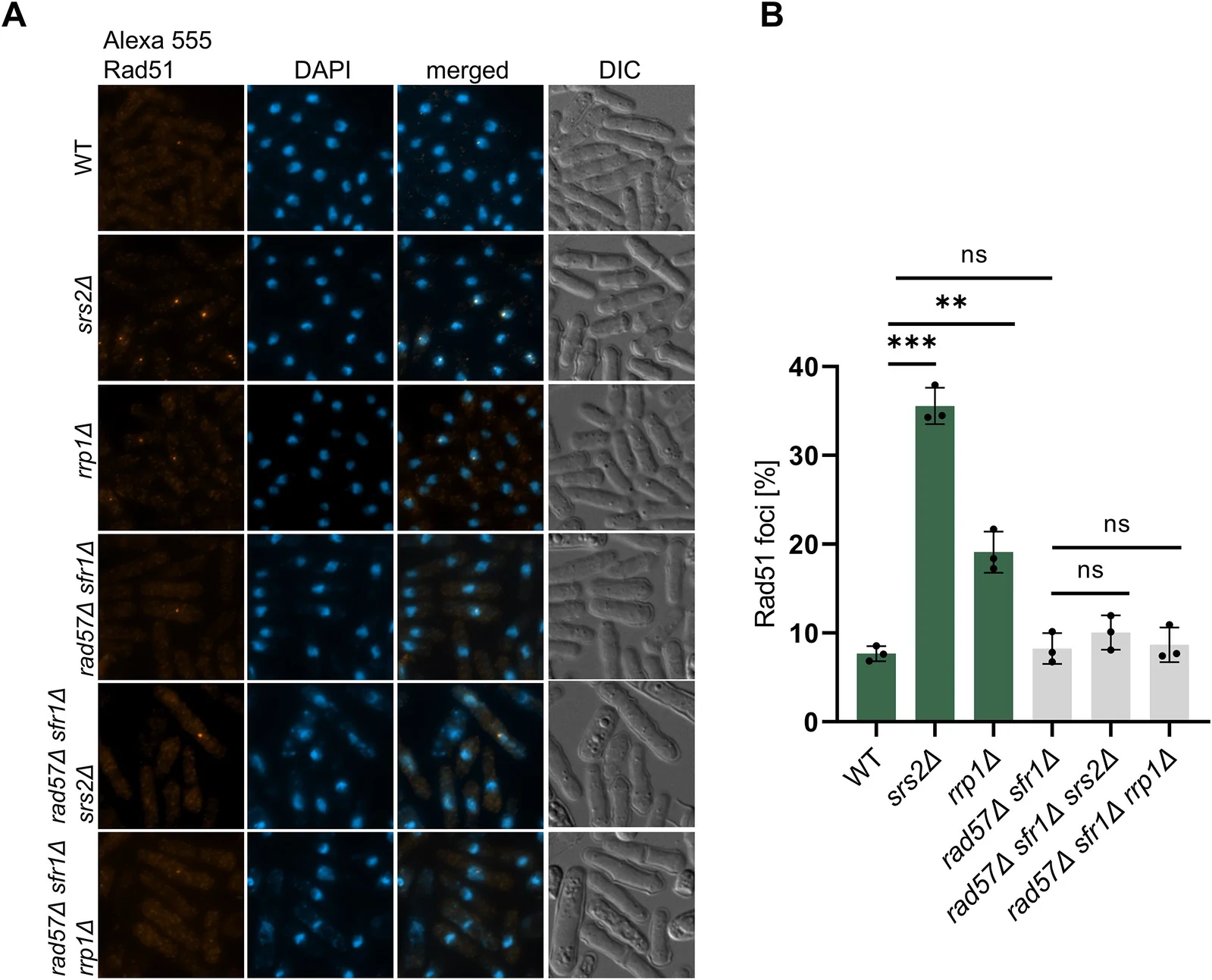
Srs2 and Rrp1 preferentially affect Rad51 nucleofilaments activated by Sfr1 and Rad57 auxiliary factors. (**A**) Representative immunofluorescence images of endogenous Rad51 protein in indicated strains. Scale bar = 5 μm. (**B**) Percentage of cells forming Rad51 foci in indicated strains. At least 400 cells were analysed for each transformant. Dots represent values for independent biological replicates. Error bars show the SD around the mean values. Two-sided Student’s *t-*test was used to calculate the *P*-values.

Surprisingly, however, we observed a significant increase in the number of Rad51-3A foci, both in S and G2 phases of the cell cycle, when *rrp1^+^*was overexpressed (Fig. 9D and E). Additional overproduction of spSrs2 had no effect, as expected. Since our data suggest that Rrp1 cannot remove Rad51-3A from chromatin, additional Rrp1 activity may lead to the generation of ssDNA substrate on which Rad51-3A can accumulate. Alternatively, increased Rrp1 activity can stabilize Rad51-3A and prevent its removal from DNA by other proteins. Since we have previously shown that Rrp1 acted as an E3 ubiquitin ligase and modified Rad51 both *in vivo* and *in vitro* [13], we set out to examine how Rrp1 affects Rad51-3A ubiquitination. We reproduced our earlier result, showing *in vivo* that ubiquitinated Rad51 accumulates in cells overproducing Rrp1, and observed that the fraction of the ubiquitinated form of strand-exchange-deficient Rad51 is larger than that of the WT protein (Supplementary Fig. S9). It is thus possible that Rrp1-stimulated ubiquitination may contribute to the increased accumulation of Rad51-3A on DNA, suggesting that, in addition to its translocase activity, Rrp1 may modulate Rad51 functions, presumably in the protection of nascent DNA. More work is clearly required to decipher the interplay between Rrp1 translocase and E3 ubiquitin ligase activities in the regulation of Rad51.

## Discussion

Regulation of proper Rad51 nucleation is a critical step to control genomic stability in eukaryotic cells. A complex interplay between helicases and translocases balances pro- and anti-recombinogenic functions of HR regulators and Rad51 nucleation. This equilibrium is crucial to avoid excessive Rad51 levels, which lead to uncontrolled Rad51 binding to dsDNA and may induce genome rearrangements. Multiple DNA helicases and translocases act to control Rad51 levels on chromatin. While toxicity of overproduced Rad51 is well documented, it has not been examined how dysregulation of enzymes acting as anti-recombinases might affect Rad51 activity.

Budding yeast Srs2 was shown to counteract nucleation of Rad51 recombinase, but also to promote error-free recombination outcomes by stimulating SDSA. In *S. cerevisiae*, a recent report underlined the necessity for restriction of scSrs2 anti-recombinase activity by Rad54 and Rdh54 motor enzymes, which limit scSrs2 disruption of Rad51 filaments [29]. Moreover, Uls1 DNA translocase from budding yeast was found to function together with Rad54 and Rdh54 in preventing the accumulation of toxic Rad51 complexes on dsDNA in *S. cerevisiae* cells [11]. Our recent findings described a role of *S. pombe* Rrp1 translocase, an Uls1 orthologue, in the regulation of Rad51 binding to dsDNA [13]. Interestingly, our initial analyses of genetic interactions placed Rrp1 together with spSrs2 helicase within the SDSA branch of HR [22]. Here we found that spSrs2 and Rrp1, when overproduced, immunoprecipitated with each other and were enriched at centromeres and rDNA. This raised the possibility that they would jointly influence Rad51 activity, in accordance with our previous models. Indeed, we observed an increase in Rad51 enrichment at the centromere locus in both *srs2*Δ and *rrp1*Δ mutants, emphasising the importance of Rrp1 activity in this region described before [13, 51]. We also confirmed that in the *srs2*Δ mutant DNA replication within rDNA was perturbed [35] and showed that the Rad51 occupancy in this region was increased. However, Rrp1 did not seem to participate in Rad51 regulation within the rDNA region as it did not affect Rad51 binding within this locus. Moreover, depletion of Rrp1 had no effect on the DNA replication through the barriers present in the rDNA locus. Increased fork stalling in rDNA region was observed only when Rrp1 was overproduced, which rather suggests its unscheduled activity.

Additionally, we have demonstrated that *S. pombe* Srs2 helicase may interact with PCNA and Rad51 like its *S. cerevisiae* orthologue and is important for the regulation of Rad51, Rad52, and RPA protein levels on DNA. Interestingly, heterologous expression of the *scSRS2* gene in fission yeast *srs2Δ* mutant was able to complement its phenotypes. This underscores the similarities between Srs2 function in the regulation of Rad51 in both yeasts. Somewhat surprisingly, we observed an accumulation of Rad51 foci not only in cells devoid of *srs2^+^*, which is expected given its well-documented ability to remove Rad51 from DNA, but also in cells overproducing spSrs2 and Fbh1, other helicase known to negatively regulate Rad51 binding to DNA [54, 63]. This suggests that the unscheduled activity of these enzymes may create a substrate for Rad51 binding. Accordingly, we have previously observed brighter RPA foci in spSrs2-overproducing cells in *S. pombe* [35], indicating that an excess of ssDNA was formed. In humans, FBH1 was also found to facilitate the generation of ssDNA at replication forks stalled by HU treatment [26]. In fission yeast, a slight increase in RPA foci has previously been shown in untreated cultures upon overexpression of *fbh1^+^* [27] suggesting that it may also create a substrate for RPA and subsequent Rad51 binding, as proposed above for spSrs2. Altogether, this could account for the accumulation of Rad51 foci that we observed in cells overproducing these helicases in untreated conditions.

We found that Rrp1 does not target Rad51 accumulating on DNA when *srs2^+^* was absent or overexpressed. Moreover, in the double *srs2*Δ *rrp1*Δ mutant, the increase in the number of Rad51 foci on DNA is greater than in the respective single mutants, indicating that both proteins may affect different pools of Rad51, located at different chromatin regions and/or bound to ss- or dsDNA. Studies in budding yeast have demonstrated that toxicity induced by Rad51 overproduction was associated with the increased accumulation of Rad51 foci on undamaged chromatin and was aggravated in cells devoid of three dsDNA translocases: Rad54, Rdh4, and Uls1 [11]. In human cells, it has also been found that upon *RAD51* overexpression, numerous foci and long fibres accumulated without associated RPA foci, suggesting that overproduced RAD51 accumulated on undamaged chromatin, at sites that do not contain ssDNA [12]. Rad54-family translocases have been shown to counteract such excessive binding of recombinase to dsDNA. As described earlier, we have previously found that in *S. pombe* Rrp1, an Uls1 orthologue, counteracts the toxicity of Rad51 overproduction and removes Rad51 fibres, establishing Rrp1 as a dsDNA translocase [13]. Here we have shown that spSrs2, in contrast to Rrp1, is unable to remove Rad51 bound to dsDNA in the form of long fibres and to rescue the growth defect resulting from excessive accumulation of Rad51 recombinase on DNA. Fbh1 also did not have the ability to alleviate the toxic effects of Rad51 overproduction.

Together, these results suggested that spSrs2 and Rrp1 might target different Rad51 species, and we decided to examine their nature in more detail. First, spontaneous Rad51 foci that accumulated both upon depletion and overexpression of *srs2^+^* helicase were dependent on the presence of the HR mediator Rad52. We thus hypothesize that they represent canonical Rad51 nucleofilaments. Overproduction of Rrp1 did not result in a decrease in the number of Rad51 foci in *rad52*Δ, suggesting that initial Rad51 loading on ssDNA by Rad52 was required before it could be removed by Rrp1.

We then showed that Rad51-3A, deficient in strand-exchange activity, cannot be removed from DNA by either spSrs2 or Rrp1, suggesting that both proteins engage with activated Rad51 nucleofilaments. It has been demonstrated in *S. pombe* that the two auxiliary complexes Rad55–Rad57 and Swi5–Sfr1 activate the Rad51 presynaptic filament [59], and in cells devoid of those complexes Rad51-mediated strand exchange is compromised. We thus examined the activity of spSrs2 and Rrp1 in these mutants and we found that deletion of *rrp1^+^*or *srs2^+^*in the background of single *rad57*Δ and *sfr1*Δ and double *rad57*Δ *sfr1*Δ mutants also had no effect on the number of Rad51 foci. This agrees with our results for the *rad51-3A* mutant and corroborates in a different system that Rrp1 and Srs2 may affect only strand-exchange-proficient forms of Rad51.

We also examined the effect of upregulation of spSrs2 and Rrp1 in the *rad51-3A* mutant, and for spSrs2 we observed no accumulation of Rad51-3A mutant on DNA, consistent with the hypothesis that spSrs2 preferentially targets activated Rad51 nucleofilaments. Unexpectedly, however, the overproduction of Rrp1 in *rad51-3A* mutant cells led to the accumulation of Rad51-3A in the nucleus and a marked increase in Rad51-3A ubiquitination. We have previously shown that Rrp1 can ubiquitinate WT Rad51, both *in vitro* and *in vivo*, but the role of this modification has not been assessed [13]. Fbh1 was reported as an E3 ubiquitin ligase targeting Rad51 in fission yeast and in humans [54, 55]. This modification was proposed to negatively regulate RAD51 because RAD51-K58/64R mutant, whose ubiquitination was attenuated, exhibited enhanced DNA binding during replication stress [55]. K58 and K64 are conserved in *S. pombe* and may also be targets of Fbh1 activity. However, a total of 15 lysines lie within Rad51 domains that constitute potential sites for modifications, so their ubiquitination by other E3 ligases, such as Rrp1, may have a different effect on Rad51 binding to DNA, and/or protect it from removal by helicases/translocases, particularly if the function of Rad51 is compromised by additional mutations.

In summary, we present genetic data indicating that spSrs2 and Rrp1 may preferentially regulate separate pools of Rad51 molecules, bound to ss- and dsDNA, respectively, but interestingly both can only remove the recombinase from DNA when the nucleofilament is activated. This suggests that spSrs2 and Rrp1 may regulate Rad51 after it has engaged in strand exchange, at ssDNA during D-loop formation and at ss-/dsDNA during replication fork protection and remodelling, to channel the repair into a specific pathway and/or restrict the generation of toxic non-productive intermediates. It is worth noting, however, that direct mechanistic proof of our hypothesis would require biochemical analyses of the activity of purified Rrp1 and/or Srs2 proteins on nucleofilaments of WT and strand-exchange-deficient Rad51 assembled on ss- or dsDNA. Additional regulators might engage Rad51 at other stages in these processes. This is exemplified by a recent work documenting in detail the role of Rad54 in assisting the Rad51 nucleofilament in homology search, synaptic complex assembly and later during D-loop formation, while simultaneously actively limiting non-productive heterologous contacts [64, 65]. Our data show that overproduction of Rrp1 increases genomic occupancy of strand-exchange-deficient Rad51. This suggests that when Rad51 binds DNA at the sites of endogenous replication stress as an inactive nucleofilament, Rrp1 may induce formation of potentially deleterious structural intermediates, promote further Rad51 binding, and engage factors involved in Rad51 post-translational modification. Further studies are needed to elucidate this aspect of Rrp1 activity and the interplay between spSrs2, Fbh1, and Rrp1 in Rad51 recombinase regulation.

## Supplementary Material

gkag978_Supplemental_Files

## Data Availability

The data underlying this article are available in the article and in its online supplementary material.
