# Supplementary material for: Distinct ways by which Srs2 helicase and Rrp1 translocase shape Rad51–DNA interactions in fission yeast
Source: Nucleic Acids Res. 2026 Oct 13;54(19):gkag978. doi: 10.1093/nar/gkag978 (PMC13659241; doi:10.1093/nar/gkag978)
Supplement: Supplementary file 1 [file gkag978_supplemental_files.zip › Supplementary-Data.pdf]

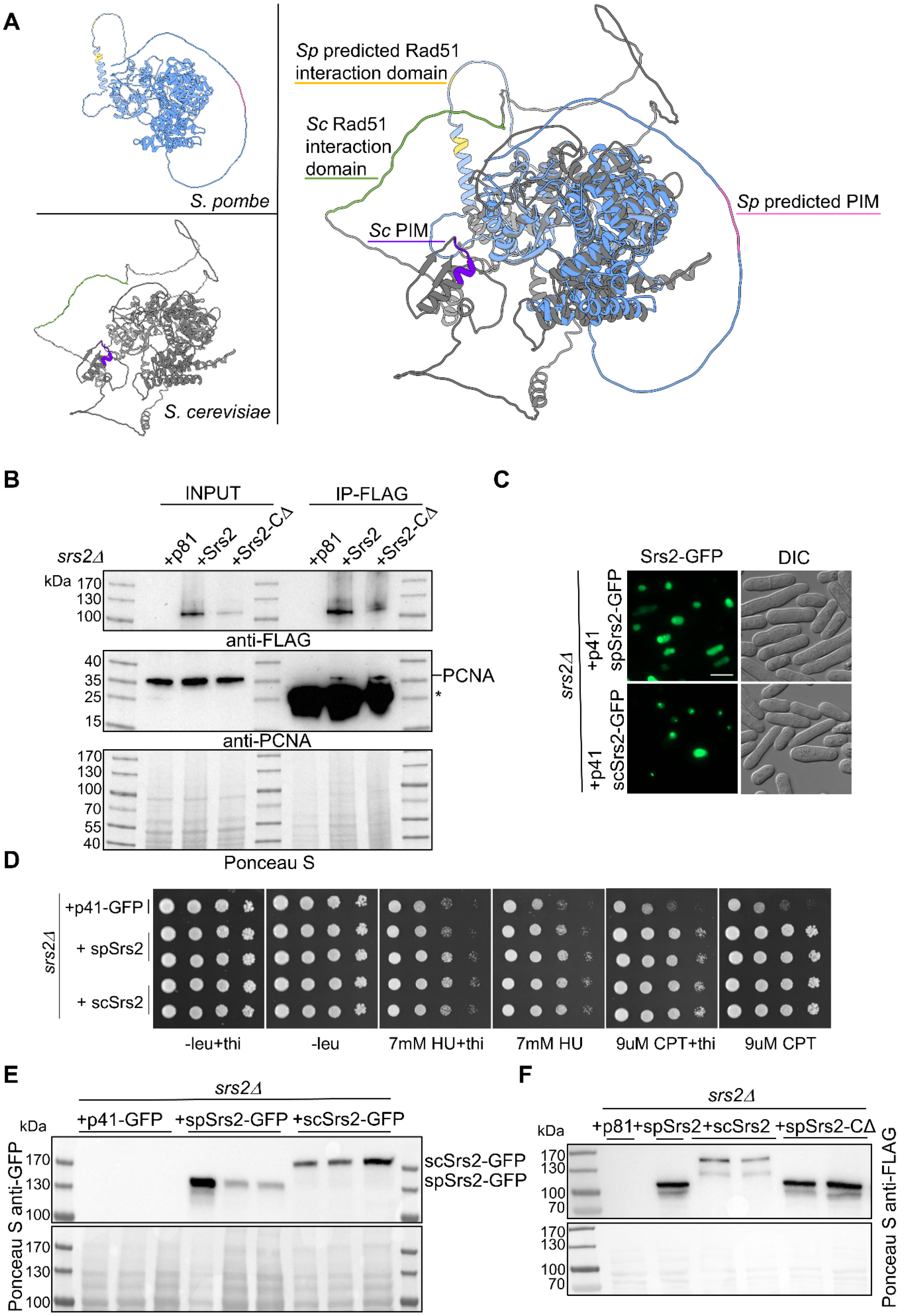

Supplementary Figure 1. Comparison between spSrs2, spSrs2-CΔ and scSrs2. A AlphaFold comparison of Srs2 structures derived from *S. cerevisiae* and *S. pombe*

orthologues. **B** Co-immunoprecipitation of spSrs2-FLAG or truncated spSrs2-CΔ-FLAG(1-877) and endogenous PCNA. Native cell extracts were precipitated with anti-FLAG antibody and resolved on Western blot, *srs2Δ* strain transformed with pREP81-FLAG was used as control. Membrane was cut and upper part was developed with anti-FLAG, whereas bottom part was developed with anti-PCNA. Ponceau S is enclosed as loading control. **C** Representative fluorescent microscopy images of spSrs2-GFP and scSrs2-GFP. Scale bar = 5 µm **D** Drop dilution assay of *srs2Δ* strain transformed with pREP41-spSrs2-GFP, pREP41-scSRS2-GFP or pREP81-GFP plasmids on indicated drugs. Cells were appropriately diluted and spotted on EMM plates containing HU or CPT, with or without thiamine (to suppress or induce plasmid expression, respectively). **E** Western blot of total cell extracts presenting levels of scSrs2-GFP or spSrs2-GFP in analysed transformants. Ponceau S is included as loading control. **F** Western blot of total cell extracts presenting levels of scSrs2-FLAG, spSrs2-FLAG and spSrs2-CΔ-FLAG in analysed transformants, *srs2Δ* strain transformed with pREP81-FLAG was used as control. Ponceau S is shown as a loading control.

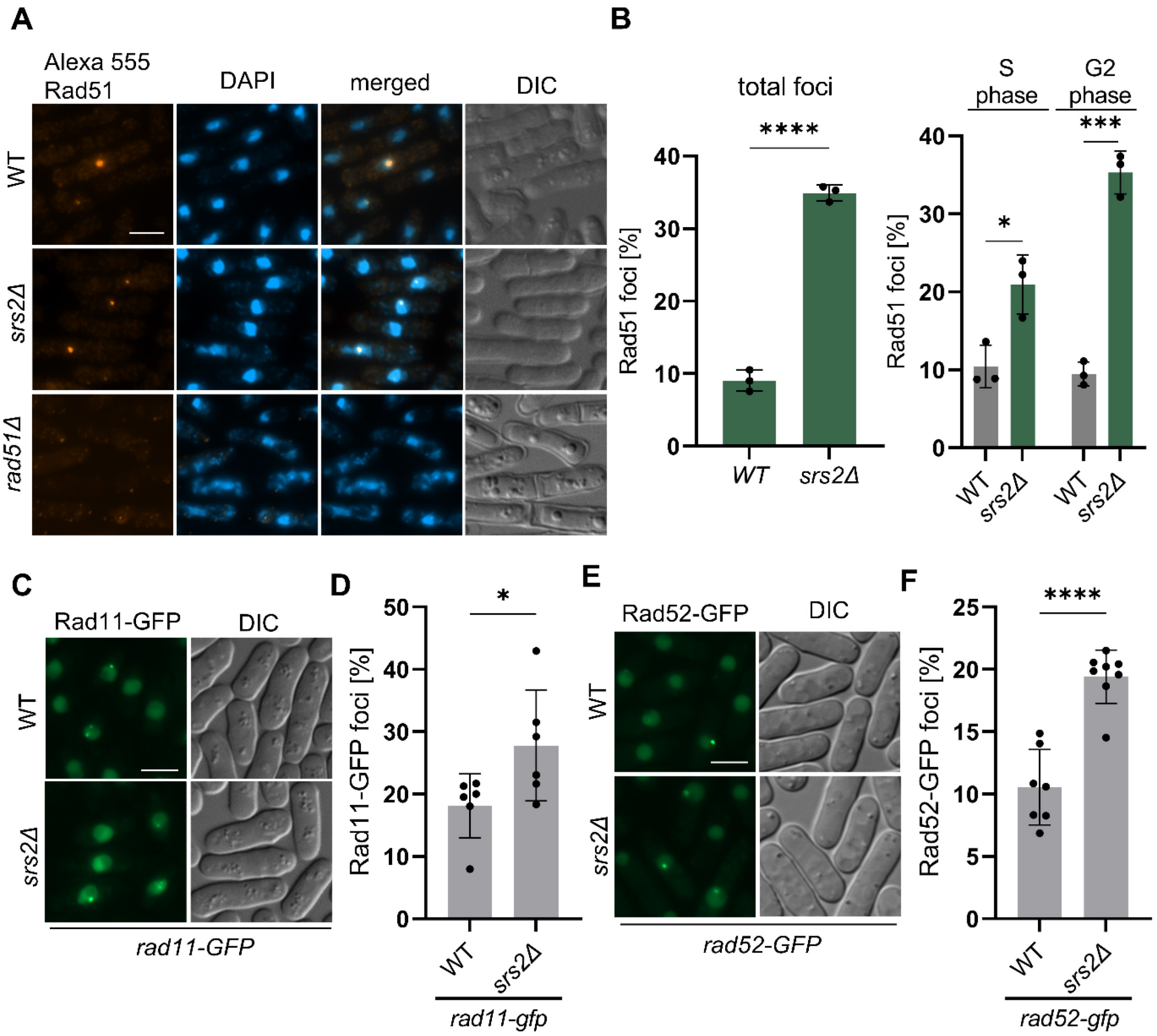

Supplementary Figure 2. Anti-recombinogenic features of fission yeast Srs2. A Representative immunofluorescence images of endogenous Rad51 protein in indicated strains. *rad51Δ* strain included as specificity control for immunochemistry. Scale bar = 5 µm **B**

Percentage of cells forming Rad51 foci in indicated strain. Left panel: total Rad51 foci, right panel: distribution of Rad51 foci in S and G2 phase. Around 1000 cells were analysed for each strain. Dots represent values for independent biological replicates. Error bars show the SD around the mean values. Two-sided Student's *t*-test was used to calculate the *p*-values. **C** Representative fluorescent microscopy images of Rad11-GFP in indicated strains. Scale bar = 5 μm **D** Percentage of cells forming Rad11-GFP foci in indicated strains. At least 400 cells were analysed for each strain. Dots represent values for independent biological replicates. Error bars show the SD around the mean values. Two-sided Student's *t*-test was used to calculate the *p*-values. **E** Representative fluorescent microscopy images of Rad52-GFP in indicated strains. Scale bar = 5 μm **F** Percentage of cells forming Rad52-GFP foci in indicated strains. At least 400 cells were analysed for each strain. Dots represent values for independent biological replicates. Error bars show the SD around the mean values. Two-sided Student's *t*-test was used to calculate the *p*-values.

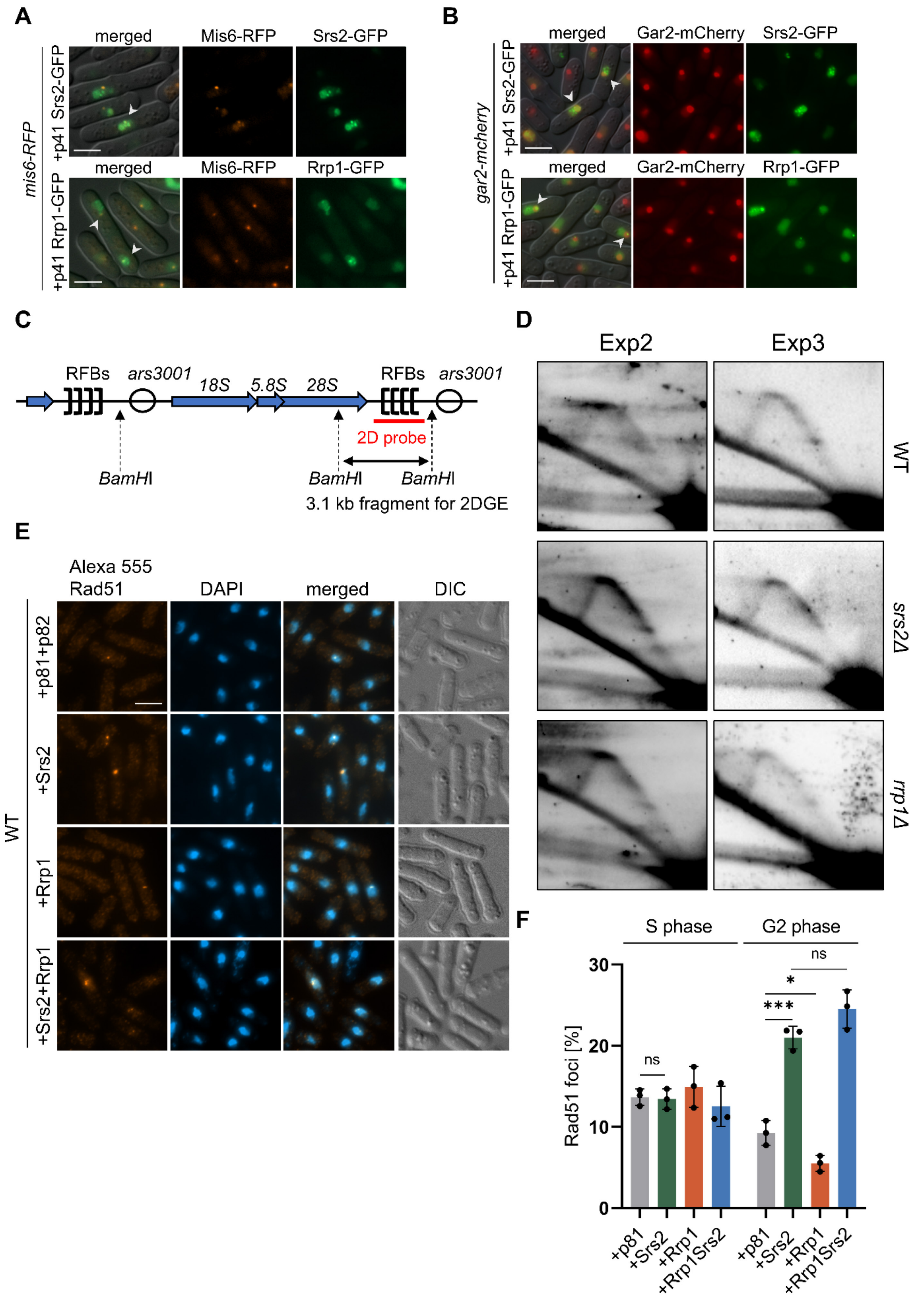

Supplementary Figure 3. Localization of overproduced Srs2 and Rrp1 and their influence on creation of Rad51 foci. A-B Representative fluorescent microscopy images of

co-localization between Srs2-GFP or Rrp1-GFP with centromere visualized by Mis6-RFP or rDNA visualized by Gar2-mCherry, respectively. Scale bar = 5 µm **C** Diagram of the rDNA locus showing the positions of replication fork barriers (RFBs) within *BamH*I restriction fragments used for two-dimensional gel electrophoresis (2DGE) and the location of the probe used for Southern blot analysis **D** Biological replicates of 2DGE Southern blots of rDNA locus in indicated strains. Exp2-Exp3 represent the second and third experiments. **E** Representative immunofluorescence images of endogenous Rad51 protein in indicated transformants. Scale bar = 5 µm **F** Percentage of cells forming Rad51 foci in indicated transformants. Around 1000 cells were analysed for each transformant. Dots represent values for independent biological replicates. Error bars show the SD around the mean values. Two-sided Student's *t*-test was used to calculate the *p*-values.

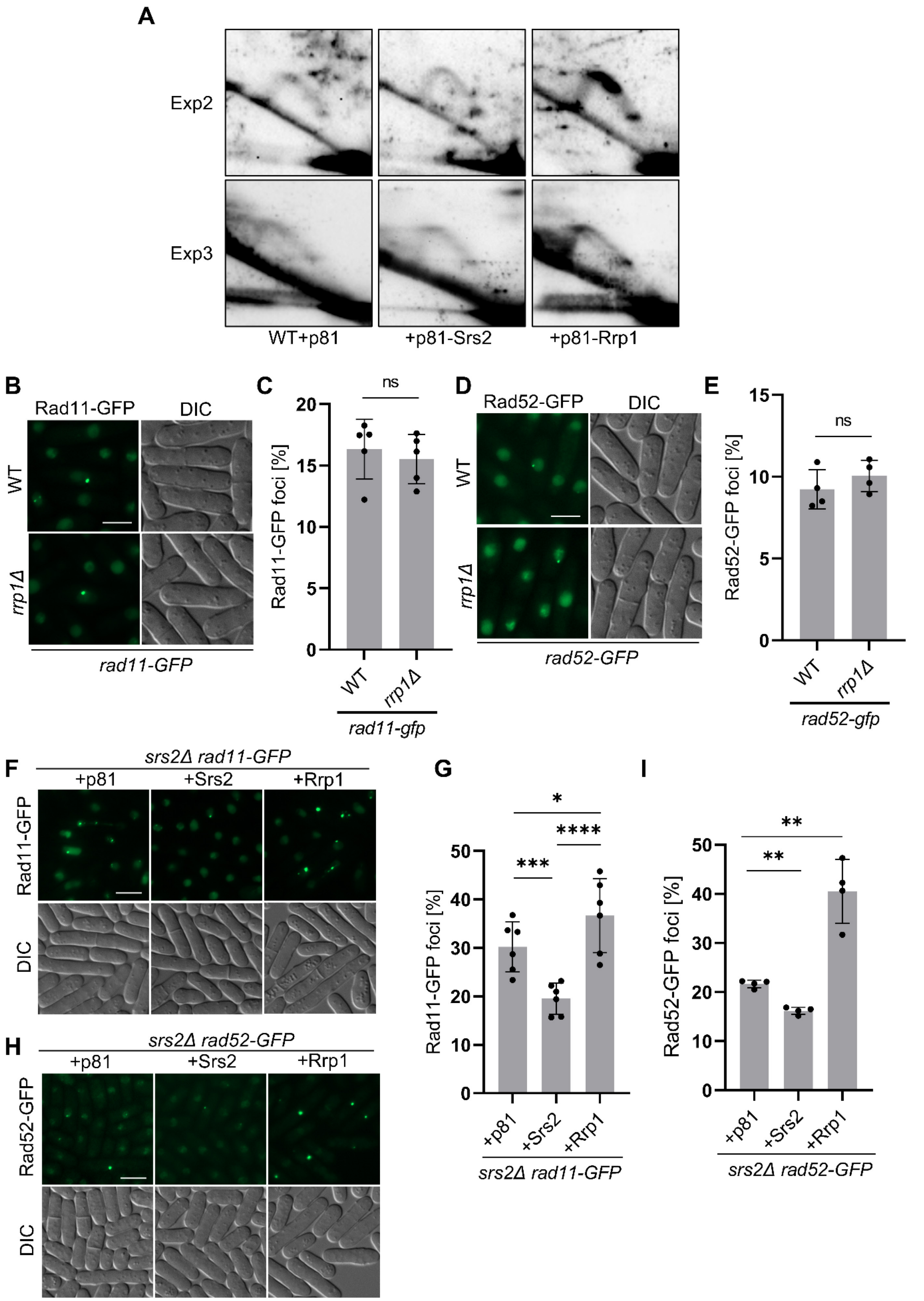

Supplementary Figure 4. Comparison of *srs2*+ and *rrp1*+ deletions on Rad52 and RPA foci formation. A Biological replicates of 2DGE Southern blots of rDNA locus in indicated

conditions. Exp2-Exp3 represent the second and third experiments. **B** Representative fluorescent microscopy images of Rad11-GFP in indicated strains. Scale bar = 5 μm **C** Percentage of cells forming Rad11-GFP foci in indicated strains. At least 500 cells were analysed for each strain. Dots represent values for independent biological replicates. Error bars show the SD around the mean values. Two-sided Student's *t*-test was used to calculate the *p*-values. **D** Representative fluorescent microscopy images of Rad52-GFP in indicated strains. Scale bar = 5 μm **E** Percentage of cells forming Rad52-GFP foci in indicated strains. At least 500 cells were analysed for each strain. Dots represent values for independent biological replicates. Error bars show the SD around the mean values. Two-sided Student's *t*-test was used to calculate the *p*-values. **F** Representative fluorescent microscopy images of Rad11-GFP in indicated transformants. Scale bar = 5 μm **G** Percentage of cells forming Rad11-GFP foci in indicated transformants. At least 400 cells were analysed for each transformant. Error bars show the SD around the mean values. Two-sided Student's *t*-test was used to calculate the *p*-values. **H** Representative fluorescent microscopy images of Rad52-GFP in indicated transformants. Scale bar = 5 μm **I** Percentage of cells forming Rad52-GFP foci in indicated transformants. At least 400 cells were analysed for each transformant. Error bars show the SD around the mean values. Two-sided Student's *t*-test was used to calculate the *p*-values.

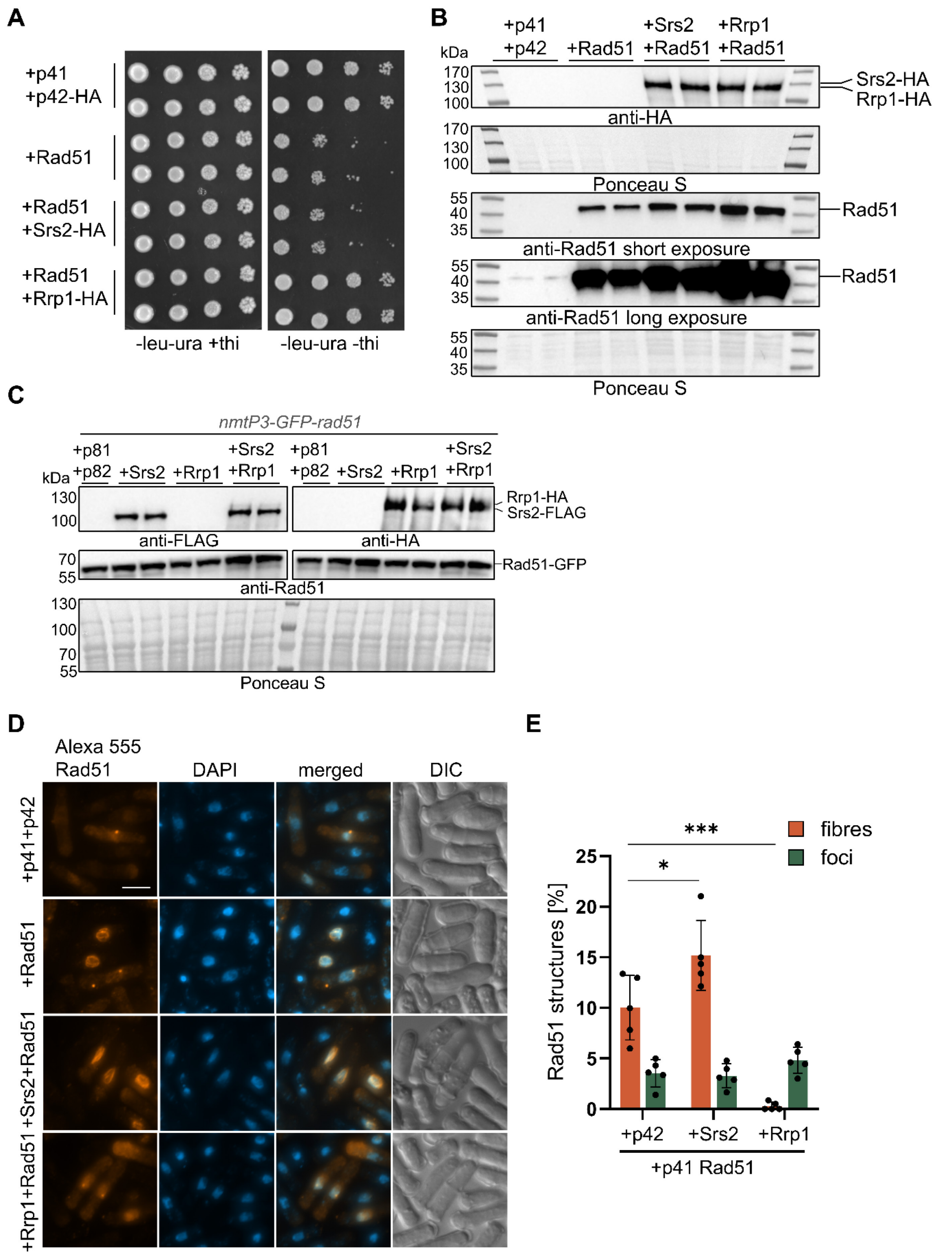

Supplementary Figure 5. Effects of Rrp1 or Srs2 overproduction in cells overexpressing untagged *rad51*+. A Drop dilution assay of WT strain transformed with indicated plasmids in conditions with repressed (+thi) or induced (-thi) expression. **B** Western blot of total cell extracts presenting levels of Srs2-HA, Rrp1-HA and Rad51 in analysed transformants. A longer exposure is included to visualize native levels of Rad51 in WT strains transformed with

empty vectors pREP41, pREP42. Ponceau S is included as loading control. **C** Western blot of total cell extracts presenting levels of Rrp1-HA, Srs2-FLAG and GFP-Rad51 in the analysed transformants. The membrane was cut: the upper left part was developed with anti-FLAG, the upper right part was developed with anti-HA, and the lower part with anti-Rad51. Ponceau S is included as a loading control. **D** Representative immunofluorescence images of overproduced untagged Rad51 protein in indicated transformants. Scale bar = 5 μm **E** Percentage of cells forming Rad51 foci and fibers in indicated transformants. Around 500 cells were analysed for each transformant. Dots represent values for independent biological replicates. Error bars show the SD around the mean values. Two-sided Student's *t*-test was used to calculate the *p*-values.

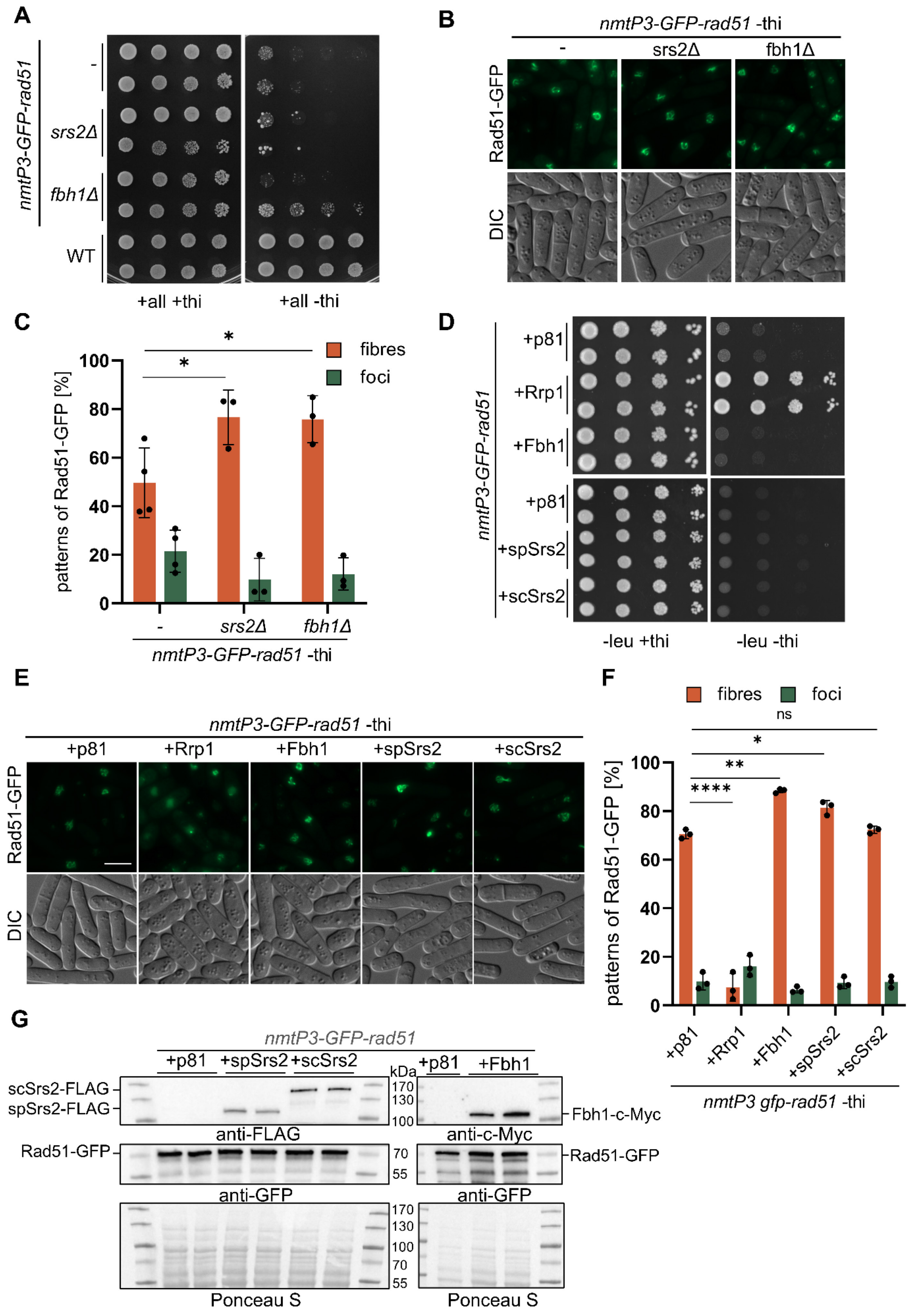

Supplementary Figure 6. Comparison of *scSRS2*, *spsrs2+*, *fbh1+* and *rrp1+* overexpression effects in cells constitutively overproducing GFP-Rad51. A Drop dilution

assay of *nmtP3-GFP-rad51*, *nmtP3-GFP-rad51 srs2Δ* and *nmtP3-GFP-rad51 fbh1Δ* strains in conditions with repressed (+thi) or induced (-thi) expression. **B** Representative images of GFP-Rad51 structures in indicated strains upon induction of expression (-thi). Scale bar = 5 µm **C** Percentage of overproduced Rad51 patterns in indicated strains (-thi). At least 400 cells were analysed. Dots represent independent biological replicates. Error bars show the SD around the mean values. Two-sided Student's *t*-test was used to calculate the *p*-values. **D** Drop dilution assay of nmtP3-GFP-rad51 strain transformed with indicated plasmids in conditions with repressed (+thi) or induced (-thi) expression. **E** Representative images of GFP-Rad51 structures in indicated transformants upon induction of expression (-thi). Scale bar = 5 µm **F** Percentage of overproduced Rad51 patterns in indicated transformants (-thi). At least 400 cells were analysed. Dots represent independent biological replicates. Error bars show the SD around the mean values. Two-sided Student's *t*-test was used to calculate the *p*-values. **G** Western blot of total cell extracts presenting levels of spSrs2-FLAG, scSrs2-FLAG, Fbh1-cMyc and GFP-Rad51 in analysed transformants. Membranes were cut and lower part was developed with anti-Rad51 and upper part with anti-FLAG or anti-cMyc. Ponceau S is included as a loading control.

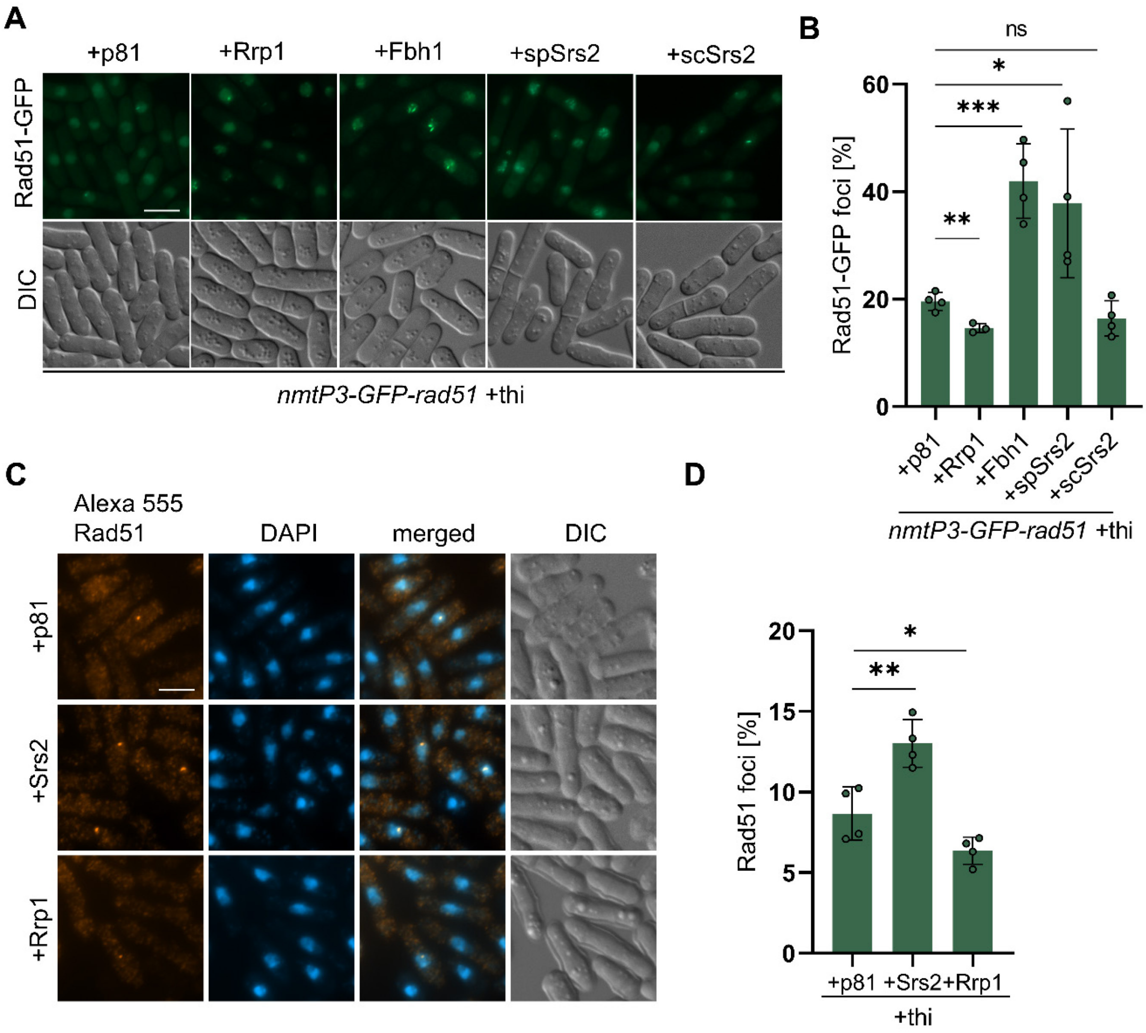

Supplementary Figure 7. Srs2 and Rrp1 act on different Rad51 pools. A Representative images of GFP-Rad51 structures in indicated transformants in repressive conditions (+thi). Scale bar = 5 µm **B** Percentage of GFP-Rad51 foci formation upon repressive conditions (+thi)

in indicated transformants. At least 400 cells were analysed. Dots represent independent biological replicates. Error bars show the SD around the mean values. Two-sided Student's *t*-test was used to calculate the *p*-values. **C** Representative immunofluorescence images of endogenous Rad51 protein in indicated transformants grown in repressive conditions (with addition of thiamine). Scale bar = 5 µm **D** Percentage of cells forming endogenous Rad51 foci in indicated strains grown in repressive conditions. At least 500 cells were analysed for each strain. Dots represent values for independent biological replicates. Error bars show the SD around the mean values. Two-sided Student's *t*-test was used to calculate the *p-values*.

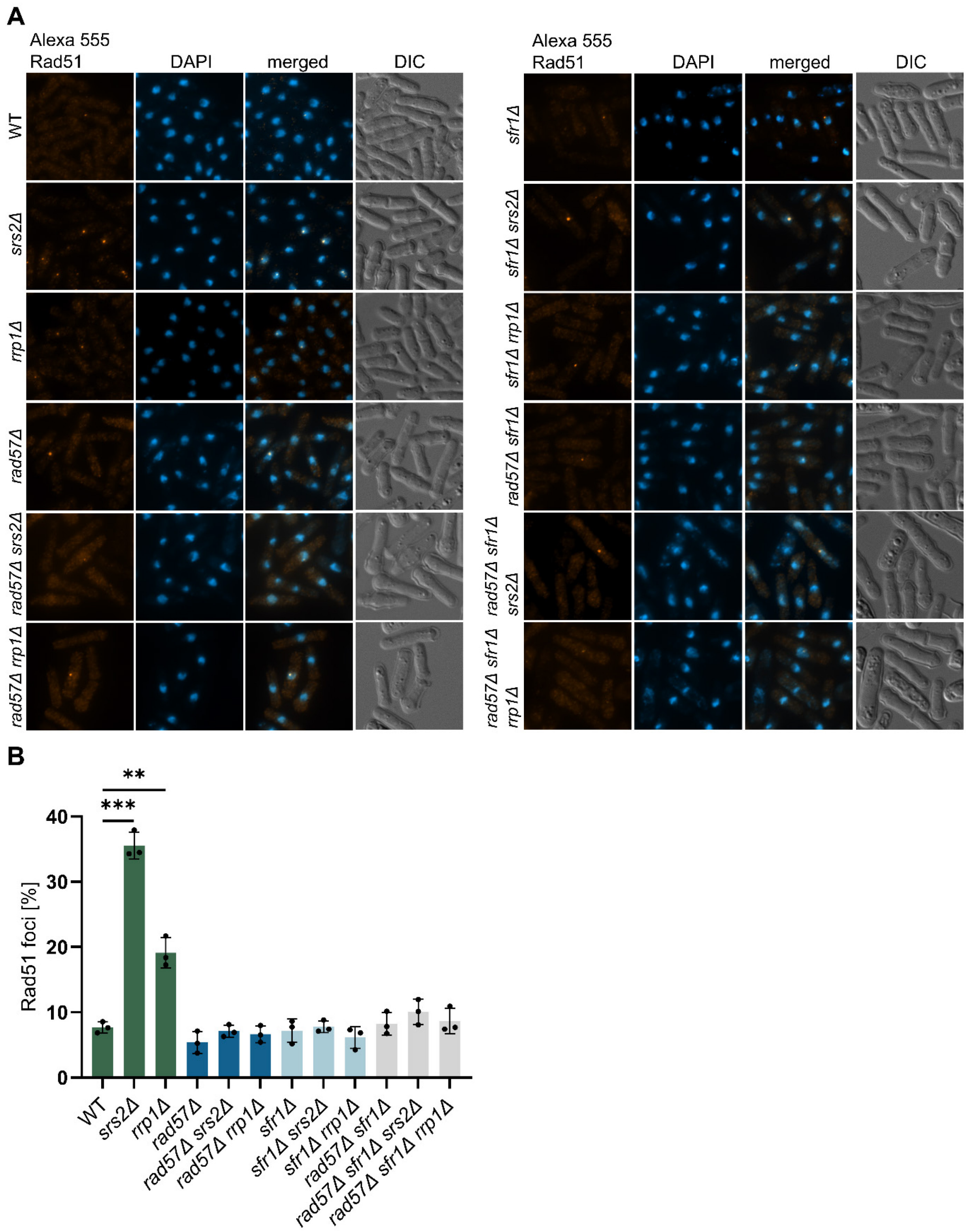

Supplementary Figure 8. Srs2 and Rrp1 act on active Rad51 nucleofilaments – complete analysis of whole set of single, double and triple mutants related to main Figure 10. A Representative immunofluorescence images of endogenous Rad51 protein in indicated strains. Scale bar = 5 µm **B** Percentage of cells forming Rad51 foci in indicated strains. At least 400 cells were analysed for each transformant. Dots represent values for independent biological replicates. Error bars show the SD around the mean values. Two-sided Student's *t*-test was used to calculate the *p-values*.

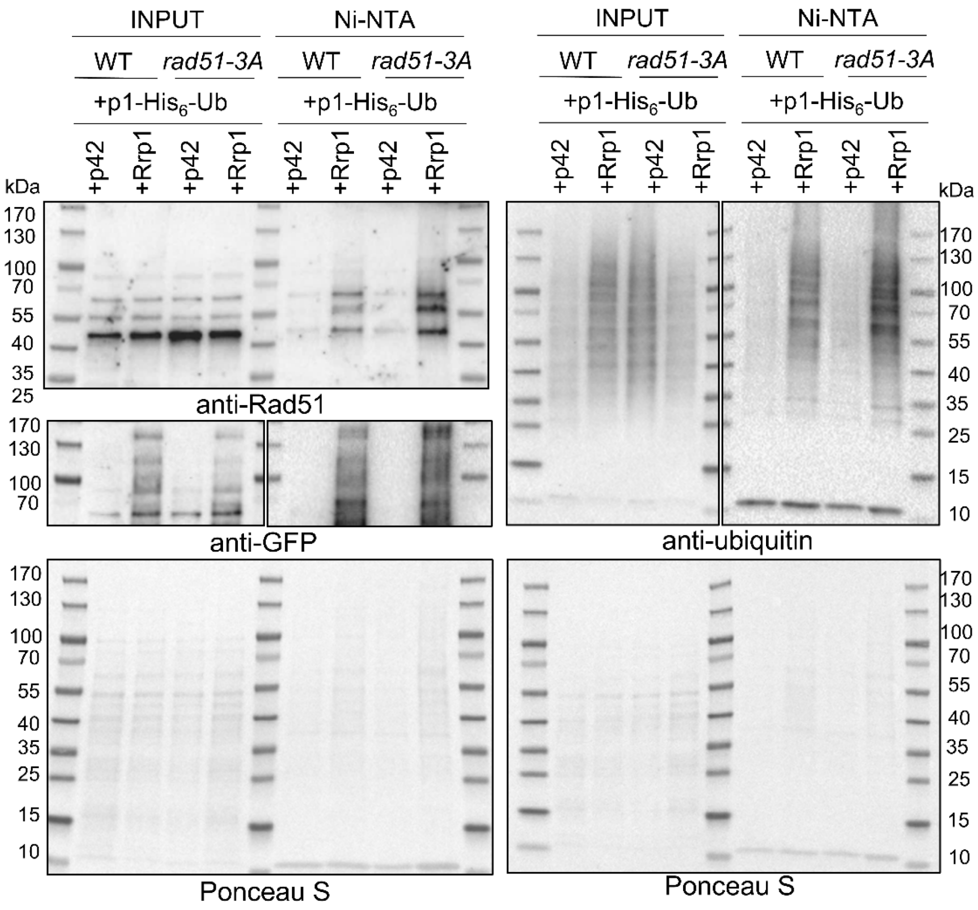

Supplementary Figure 9. Rrp1 overproduction leads to increased ubiquitination of catalytically inactive Rad51. Ubiquitination status of Rad51 and Rad51-3A by His_6-ubiquitin Ni-NTA-pulldown from WT and *rad51-3A* strains in conditions of Rrp1 overproduction. WT and *rad51-3A* were co-transformed with +pREP1-His_6-ubiquitin (marked on figure as +p1-His_6-Ub) and either pREP42-GFP-Rrp1 or empty pREP42-GFP plasmid as control. Same samples were run to obtain two membranes, left panel was probed against Rad51, then stripped and probed against GFP (two exposures are merged, full blots from each exposure presented in Supplementary Figure 12). Right panel was probed with anti-ubiquitin (two exposures were merged, full blots from each exposure presented in Supplementary Figure 12). Ponceau S is included as loading control.

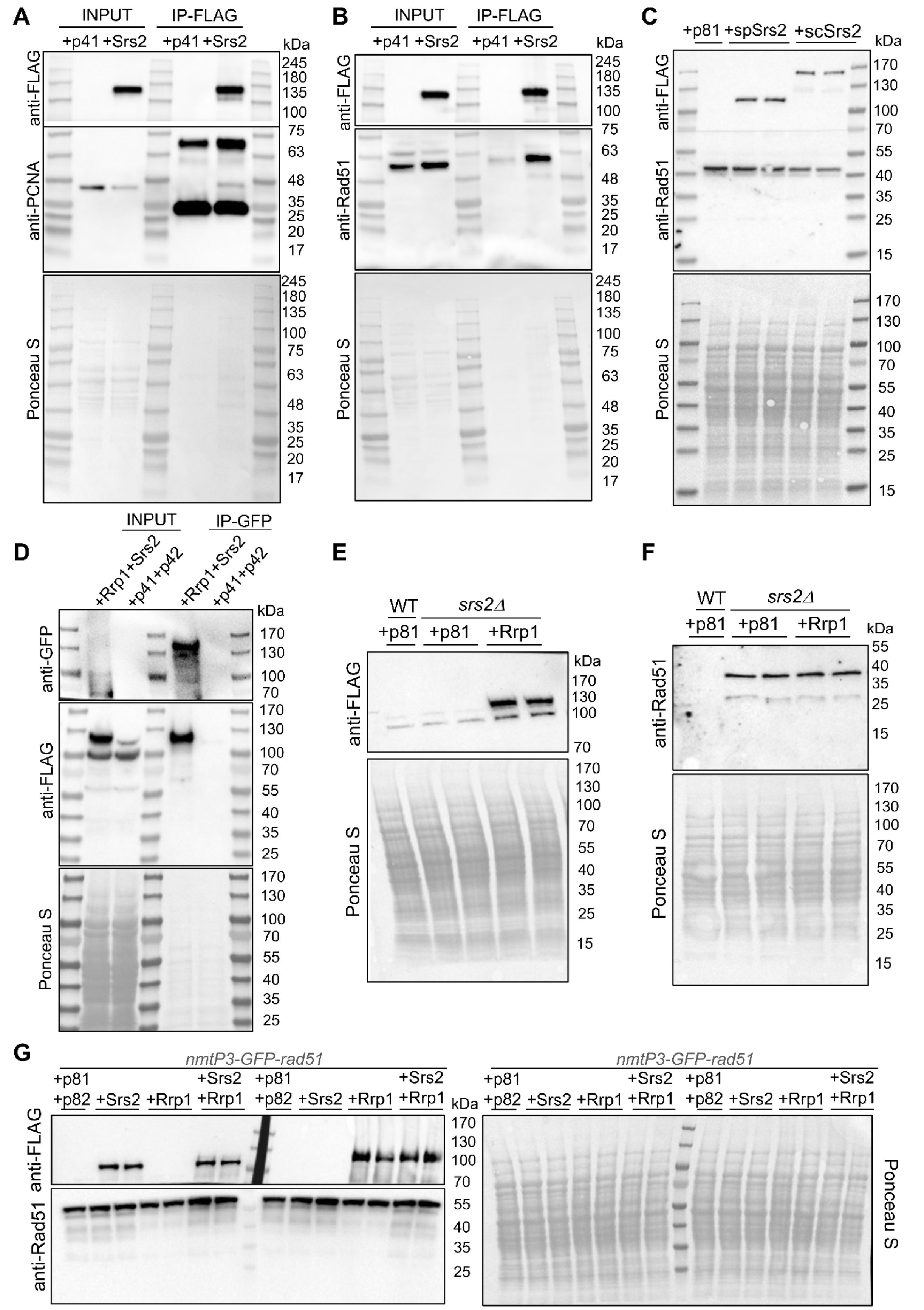

Supplementary Figure 10. Uncropped gels and blots presented in main manuscript text and Supplementary Figure 5C. A-B Source Western blots presented in Figure 1C–D. The

membranes were cut at 75 kDa and the upper and lower parts were developed with anti-FLAG and anti-PCNA (A) or anti-Rad51 (B), respectively. Ponceau S staining is shown for the intact membranes. **C** Source Western blot presented in Figure 2F. The membrane was cut at approximately 70 kDa. The upper part was developed with anti-FLAG and the lower part with anti-Rad51. Ponceau S staining is shown for the intact membrane. **D** Source Western blots for Figure 3A. The same samples were equally distributed between two parallel gels. The upper blot was developed with anti-GFP and cropped at 70 kDa, whereas the lower blot was developed with anti-FLAG. Ponceau S staining of the corresponding intact membrane is shown. **E-F** Source blots for the images presented in Figure 4D, developed with anti-FLAG (E) or anti-Rad51 (F). Ponceau S staining is shown for each blot. **G** Source Western blots for Supplementary Figure 5C. The same samples were run in duplicate. The membranes were cut horizontally at approximately 70 kDa and vertically through the molecular weight marker. The resulting membrane sections were developed as follows: the lower part with anti-Rad51, the upper left part with anti-FLAG, and the upper right part with anti-HA. Ponceau S staining of the intact membrane before cutting is shown.

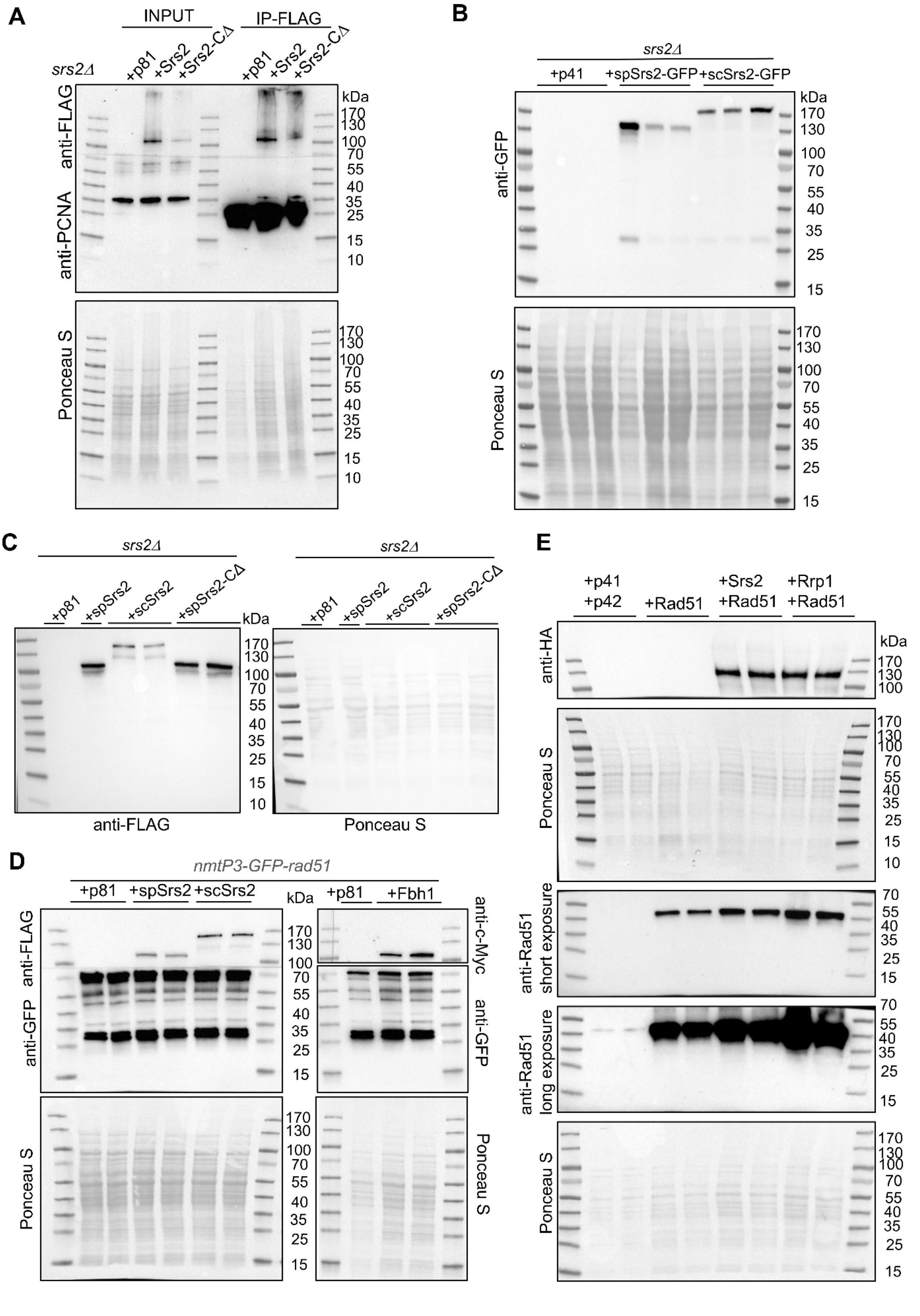

Supplementary Figure 11. Uncropped gels and blots presented in Supplementary Data file. A Source Western blots for Supplementary Figure 1B. The membrane was cut at 70 kDa. The upper part was developed with anti-FLAG and the lower part with anti-PCNA. Ponceau S

staining of the intact membrane before cropping to the relevant parts of the Western blot is shown. **B** Source anti-GFP Western blot for Supplementary Figure 1E. Ponceau S staining is shown as a loading control. **C** Source anti-FLAG Western blot for Supplementary Figure 1F. Ponceau S staining is shown as a loading control. **D** Source blots for Supplementary Figure 6G. The membranes were cut at 70 kDa. The left panel shows anti-FLAG (upper part) and anti-GFP (lower part), whereas the right panel shows anti-Myc (upper part) and anti-GFP (lower part). Ponceau S staining of the intact membranes is shown below the respective blots. **E** Source Western blots for Supplementary Figure 5B. The upper blot was developed with anti-HA, whereas the lower blot was developed with anti-Rad51; shorter and longer exposures are shown. Ponceau S staining for each Western blot is shown.

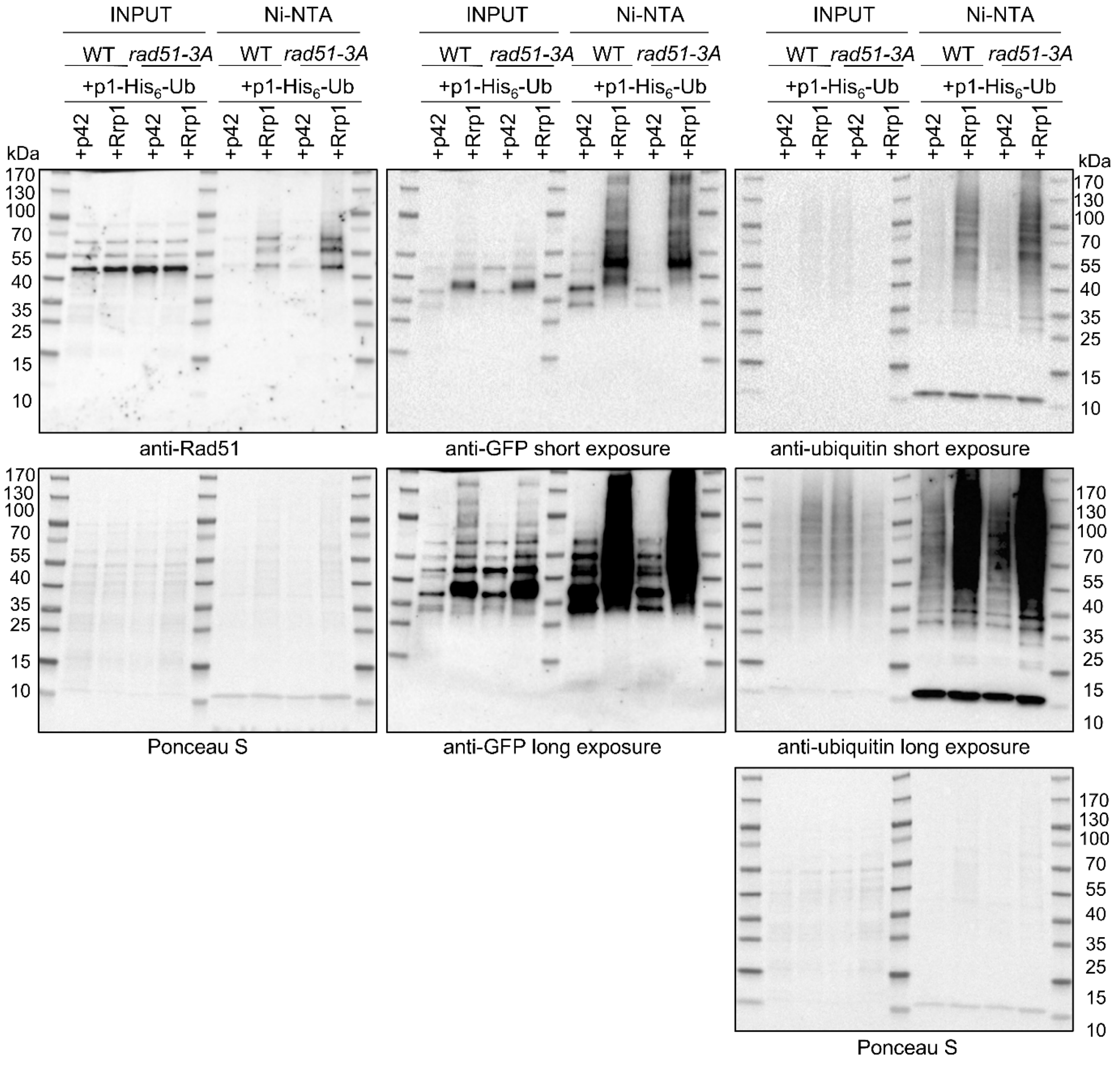

Supplementary Figure 12. Uncropped gels and blots related to Supplementary Figure 9. Source Western blots for Supplementary Figure 9. The same samples were used to prepare two membranes. The left membrane was probed with anti-Rad51, then stripped and reprobed with anti-GFP. Two exposures (shorter and longer) of the anti-GFP blot are presented in the

middle panel. Ponceau S staining shown below the anti-Rad51 blot corresponds to the same membrane used for the anti-GFP blot. The right panel shows the full anti-ubiquitin blot; two exposures (shorter and longer) are presented. Ponceau S staining is shown below as a loading control.

Supplementary Table S1: Strains and plasmids used in this study

**Strain number**	**Genotype**	**Source**
IDN1	*ade6-704 leu1-32 ura4-D18, h+*	S. Lambert
IDN33	*arg3-D1 his3-D1 leu1-32 ura4-D18 rrp1::Kan, h+*	D. Dziadkowiec
IDN37	*his3-D1 leu1-32 ura4-D18 srs2::ura4+, h+*	D. Dziadkowiec
IDN277	*his3-D1 leu1-32 ura4-D18 mis6-2mRFP:Hph, h?*	S. Lambert
IDN335	*leu1-32 ura4-D18 gar2-mCherry:Kan, h-*	NBRP
IDN620	*ade6-704 leu1-32 ura4-D18 his3-D1 rad52::Kan srs2::ura4+, h?*	this study
IDN340	*ade6-704 leu1-32 ura4-D18 rad52::Kan, h?*	S. Lambert
IDN740	*ade6-704 leu1-32 ura4::ADH1-dmNK:Nat-ADH1-hENT1, h?*	this study
IDN41	*his3-D1 leu1-32 ura4-D18 rad11-cGFP:Kan, h+*	D. Dziadkowiec
IDN188	*ade6-704 leu1-32 t-ura4-SD20-ori loxP:rad51_R152A-R324A-K334A:loxM3, h-*	S. Lambert
IDN653	*ade6-704 leu1-32 ura4-D18 his3-D1 srs2::ura4+ loxP:rad51_R152A-R324A-K334A:loxM3, h?*	this study
IDN750	*arg3-D1 his3-D1 leu1-32 ura4-D18 rrp1::Kan loxP:rad51_R152A-R324A-K334A:loxM3, h?*	this study
IDN42	*ade6-210 arg3-D1 his3-D1 leu1-32 ura4-D18 rad11-cGFP::Kan rrp1::Nat, h+*	D. Dziadkowiec
IDN44	*arg3-D1 leu1-32 his3-D1 ura4-D18 rad11-cGFP::Kan srs2::ura4+, h+*	D. Dziadkowiec
IDN523	*ade6-704 his3-D1 leu1-32 ura4-D18 srs2::ura4+ rad52-GFP:Kan, h?*	this study
IDN527	*ade6-704 his3-D1 leu1-32 ura4-D18 rad52-GFP:Kan, h?*	this study
IDN204	*ade6-704 leu1-32 ura4-D18 rad51::Kan, h?*	S. Lambert
IDN793	*ade6-704 leu1-32 ura4-D18 rad52-GFP:Kan rrp1::Nat, h?*	D. Dziadkowiec
IDN790	*leu1-32 ura4-D18 nmtP3-GFP-Rad51:Kan, h?*	D. Dziadkowiec
IDN791	*leu1-32 ura4-D18 ade6-704 nmtP3-GFP-Rad51:Kan fbh1::Nat, h?*	this study
IDN792	*leu1-32 ura4-D18 ade6-704 his3-D1 nmtP3-GFP-Rad51:Kan srs2::ura4+, h?*	this study
IDN183	*ade6-210 arg- his3-D1 leu1-32 ura4-D18 rad57::his3, h?*	D. Dziadkowiec
IDN837	*arg3-D1 his3-D1 ura4-D18 rad57::his3+ srs2::ura4+, h?*	D. Dziadkowiec

IDN839	*arg3-D1 his3-D1 ura4-D18 leu1-32 rad57::his3+ sfr1::arg3+, h?*	D. Dziadkowiec
IDN841	*ade6-704 arg3-D1 his3-D1 leu1-32 ura4-D18 rad57::his3+ sfr1::arg3+ rrp1::Kan, h?*	D. Dziadkowiec
IDN844	*arg3-D1 his3-D1 ura4-D18 rad57::his3+ sfr1::arg3+ srs2::ura4+, h?*	D. Dziadkowiec
IDN847	*ade6-704 arg3-D1 his3-D1 leu1-32 ura4-D18 rad57::his3+ rrp1::Kan, h?*	D. Dziadkowiec
IDN849	*arg3-D1 his3-D1 leu1-32 ura4-D18 sfr1::arg3+, h?*	D. Dziadkowiec
IDN850	*arg3-D1 his3-D1 leu1-32 ura4-D18 sfr1::arg3+ srs2::ura4+, h?*	D. Dziadkowiec
IDN851	*arg3-D1 his3-D1 ura4-D18 sfr1::arg3+ rrp1::Kan, h?*	D. Dziadkowiec
IDN854	*arg3-D1 his3-D1 leu1-32 ura4-D18 rrp1::Kan srs2::ura4+ h?*	D. Dziadkowiec
IDN_P13	*pREP41-MSC+*	Addgene
IDN_P85	*pREP41-3flag*	D. Dziadkowiec
IDN_P99	*pREP81-srs2-3flag*	D. Dziadkowiec
IDN_P58	*pREP41-rad51*	D. Dziadkowiec
IDN_P104	*pREP81-scSRS2-3flag*	this study
IDN_P105	*pREP81-srs2-C∆-3flag*	this study
IDN_P106	*pREP41-egfp-scSRS2-3flag*	this study
P52	*pREP81-3flag*	D. Dziadkowiec
P102	*pREP41-egfp-srs2-3flag*	D. Dziadkowiec
P19	*pREP42-3HA*	D. Dziadkowiec
P108	*pREP42-3HA-srs2-3flag*	D. Dziadkowiec
P5	*pREP41-egfp*	D. Dziadkowiec
P6	*pREP41-egfp-rrp1*	D. Dziadkowiec
P20	*pREP82-3HA-rrp1*	D. Dziadkowiec
P64	*pREP81-rrp1-3flag*	D. Dziadkowiec
P150	*pREP41-fbh1-cMyc*	M. Whitby
P135	*pREP1-6his-ubiquitin*	D. Dziadkowiec

Supplementary Table S2: Primers used in this study

**Primer name**	**Sequence (5'-3')**	**Experiment**
II50_F	CACCGCAGTTCTACGTATCCT	ChIP (Control locus on ChrII)
II50_R	CGATGTAACGGTATGCGGTA	ChIP (Control locus on ChrII)
cnt_F	CAACCGTTGCAACTTACATCAGCA	Srs2, Rrp1 and Rad51 ChIP
cnt_R	CCGGTCGCCAAATAGCAATGAGAT	Srs2, Rrp1 and Rad51 ChIP
dg_F	TACCGTGATTAGCCTTACTCCGCA	Srs2 and Rad51 ChIP
dg_R	ACCGCAAGATAGAGTAGGATGGGT	Srs2 and Rad51 ChIP
rdna_F	GGACGGTGGCCATGGAA	Rrp1 and Rad51 ChIP
rdna_R	CATTCGGCCGGTGAG	Rrp1 and Rad51 ChIP
KK146	GAATTCGGTAAGCGTTGGATTG	Southern rDNA
KK147	GAATTCTTCTTTCACATC	Southern rDNA
